# Supplementary material for: Measurement characteristics and genome-wide correlates of lifetime brain atrophy estimated from a single MRI
Source: Nat Commun. 2025 Jul 21;16:6725. doi: 10.1038/s41467-025-61978-6 (PMC12280159; doi:10.1038/s41467-025-61978-6)
Supplement: Supplementary file 1 — Supplementary Information [file 41467_2025_61978_MOESM1_ESM.pdf]

Supplementary Materials

Fürtjes et al., 2025, Measurement characteristics and genome-wide correlates of lifetime brain atrophy estimated from a single MRI
(Nature Communications)

Contents

Supplementary information for the Introduction 2

Description of visual rating scales 2

Box S1. Computational approaches used to infer lifetime brain atrophy (LBA) from either a single cross-sectional MRI scan, or observed atrophic changes from two repeated longitudinal MRI scans3

Supplementary Methods 4

Deviations from the pre-registration 4

Restricting the HCP cohort to below 31-year-olds 4

Pre-registered Aim 2; sample specificity of LBA norms and the role of ICV 7

Moderation analysis 7

GWAS re-analysis to test whether results change when adjusting for fewer genetic PCs 8

Supplementary Results 8

Repeated LBA measures increase with chronological age in the LBC1936 8

Aim 3: Associations with ageing-related variables 10

Aim 3.1: Amplifier effect 10

Aim 3.2: Does lifetime atrophy (LBA) explain variance above and beyond baseline variables TBV and ICV? 12

Aim 3.3: Does lifetime brain atrophy explain health phenotype-associated variance above and beyond the variance explained by estimated atrophy? 13

Aim 3.4: General trends of associations across aims 3.1-3.3 14

SNP-by-age interaction effects on LBA 16

GWAS re-analysis adjusting for 8 genetic PCs only (instead of 40 genetic PCs as pre-registered). 16

Supplementary plots 23

Comparing residual (two-step approach) and adjustment method (one-step approach) 48

MRI processing robustness checks in LBC1936 50

Supplementary information for the Introduction

Description of visual rating scales

There are already-available approaches for quantifying lifetime brain atrophy (LBA) from a single MRI scan. For example, in neuroradiological settings, visual rating scales aid identifying atrophy based on the extent of visible features from a single scan. Well-validated visual scales rate medial temporal lobe atrophy (MTA; Scheltens et al., 1995; Scheltens et al., 1992), global cortical atrophy (GCA; Pasquier et al., 1996), and posterior atrophy (PA; Koedam et al., 2011). In research settings, computational approaches to measuring total brain atrophy from a single MRI scan are common (Bu et al., 2021; Good et al., 2002; Kilsdonk et al., 2015). Computational approaches concur with visual rating scales, but offer greater statistical power since they capture more interindividual variability (not being restricted to a smaller – and more practical – ordinal scale), and offer comparatively greater fidelity to index subtle adulthood atrophic changes in non-clinical samples (Velickaite et al., 2020). Note that evaluations based on visual rating scales in LBC1936 showed that ratings improved over time because the raters had seen more and more older brains over the course of doing the rating which shifted their implicit baseline of what atrophy looks like. This may explain why LBA $_{difference}$ was most strongly associated with the rating scales in LBC1936 (Section 1) even though it is so strongly correlated with ICV ($r = 0.81$; *Fig.S4*).

Box S1. Computational approaches used to infer lifetime brain atrophy (LBA) from either a single cross-sectional MRI scan, or observed atrophic changes from two repeated longitudinal MRI scans

**Method**	**Interpretation**	**LBA** (inferred from a single cross-sectional MRI scan**)**	**Observed atrophic changes (**inferred from two repeated longitudinal MRI scans**)**	**Advantages**	**Disadvantages**
Difference score	Captures absolute brain matter losses that occurred since the brain maximally filled the skull<br><br>Larger values denote greater brain atrophy in mm^3	**LBA** $_{\textbf{difference}}$ = *ICV - TBV*	**Observed atrophic changes** $_{\textbf{difference}}$ = TBV_{time1} - TBV_{time2}	Intuitive to interpret in mm^3<br><br>Each participant's value can be computed independently of the study sample	Difference scores where one value is subtracted from the other are correlated with the initial value by construction (e.g., Clifton & Clifton, 2019)<br><br>Difference scores conflate the unreliability of the two contributing measures (Cronbach & Furby, 1970; Lord, 1956) particularly in instances where baseline and subsequent measures are moderately-to-strongly correlated, such as TBV and ICV (Rogosa & Willett, 1983). Thus, we expect that *ICV minus TBV* may largely index baseline levels of brain size – as opposed to brain changes only – and any resultant GWAS of this measure may mainly reflect SNPs related to head size.
Ratio score	Captures proportional brain matter losses from when TBV maximally filled the skull<br><br>Sometimes referred to as the *parenchymal volume fraction*, or *brain parenchymal fraction* (Rudick et al., 1999)<br><br>Smaller values denote greater brain atrophy*	**LBA** $_{\textbf{ratio}}$ = *TBV / ICV*	**Observed atrophic changes** $_{\textbf{ratio}}$ = TBV_{time2} / TBV_{time1}	Easy to interpret as proportional values between 0 and 1<br><br>Each participant's value can be computed independently of the study sample	Ratio scores also suffer from a mathematical coupling issue (Archie, 1981). This means, just as with difference scores (above), the ratio score of atrophy is likely correlated with ICV, and hence captures baseline differences in brain size, in addition to proportional brain matter losses
Residual score	A residual score captures the difference between an individual's observed TBV and their predicted TBV given their ICV size (i.e., a negative value means an individuals' TBV is smaller than expected given their ICV)<br><br>Smaller values denote greater brain atrophy*	*TBV*-associated residuals of *ICV*<br><br>**LBA** $_{\textbf{residual}}$ = lm(TBV ~ ICV)	TBV_{time2} -associated residuals of TBV_{time1}<br><br>**Observed atrophic changes** $_{\textbf{residual}}$ = **lm(**TBV_{time2} ~ TBV_{time1}**)**	Residual scores are uncorrelated with baseline differences in head size (i.e., ICV in single-occasion MRI estimation or TBV_{time1} in longitudinal estimation)	Residual scores could be categorised as less intuitive and less concrete than the two methods above<br><br>A regression-based approach means that an individual's residuals will differ as a function of the overall sample composition which dictates the regression line from which the residuals deviate. This makes the resulting residual score susceptible to sources of bias such as overfitting (when computed in small samples) and variance inflation (where the covariate is highly correlated with the exposure when used in a multivariable setting) which can affect regression estimates

* Throughout the paper, residual and ratio score values have been flipped (i.e., multiplied with -1) to enable a unified interpretation across all three methods whereby larger values denote greater brain atrophy.

Supplementary Methods

Deviations from the pre-registration

Restricting the HCP cohort to below 31-year-olds

We had pre-registered (https://osf.io/gydmw/) that young cohorts such as the HCP and MRi-Share should demonstrate no, or very weak associations between age and both ICV and LBA. In this project, we understand ICV to be constant across the lifetime, which is why we use it in this project to approximate premorbid brain size, i.e., the size of a participant's brain prior to any neurodegeneration. Indeed, there is evidence that ICV should be largely stable across the lifespan, but minor age-related changes can occur due to skull and meninges thickening (Caspi et al., 2020; Nerland et al., 2022; Royle et al., 2013). Previous studies have also delivered evidence for effects of ICV measurement method and study population, whereby older cohorts tend to have somewhat smaller skulls than younger cohorts (Caspi et al., 2020; Ma et al., 2019; Nerland et al., 2022).

Contrary to our expectation of no or negligible ICV-age correlations ($r < |0.1|$), the HCP data, as downloaded, showed a moderate ICV-age correlation of $r = -0.2$ ($p = 4.2\text{x}10^{-11}$). This ICV-age correlation was even stronger than the TBV-age correlation in the same sample ($r = 0.16$; $p = 4.9\text{x}10^{-8}$). Implausibly, age was negatively associated with more LBA in this young sample where atrophy was derived from ICV and TBV measures using three computational methods. That is, older HCP participants had less brain atrophy (i.e., larger values in the difference score, smaller values in the ratio score) than younger participants which is not sensible, and we suggest must be a sample artifact rather than an interpretable finding. Note, however, that age was uncorrelated with brain atrophy derived using the residual method where the atrophy score is independent of ICV by design (*Methods Figure 5* below).

In the process of making sense of these unexpected correlational patterns, we excluded related individuals (n = 445), and adjusted for batch effects, none of which reduced, or substantially altered either the ICV-age correlation, or the LBA-age correlation. We had anticipated in our pre-registration that there may be unexpected correlations between age and ICV (as well as the atrophy measures), which could be driven by few older-age outliers. To test for this, we successively reduced the sample age (equivalent procedure to analyses presented in *Fig.3D-G*) by applying maximum-age cut-offs to obtain a subsample of individuals in which age and ICV are negligibly correlated below our pre-registered but arbitrary cut-off of $r < |0.1|$. Indeed, the strength of the ICV-age correlation was moderated by sample age, and excluding 303 participants > 31-years reduced the ICV-age correlation to $r < 0.1$ (*Methods Figure 6 below*). This indicates a cohort effect whereby older participants in the HCP have systematically smaller skulls than younger participants. *Methods Figure 7* (below) illustrates that the HCP restricted to below 31-year-olds produces a negligible age-ICV correlation ($r < |0.1|$), as well as non-significant lifetime atrophy-age correlations, which is in keeping with our expectations for young adults (outlined in the pre-registration). Hence, all analyses presented in the main manuscript were calculated in the HCP participants below the age of 31 years.

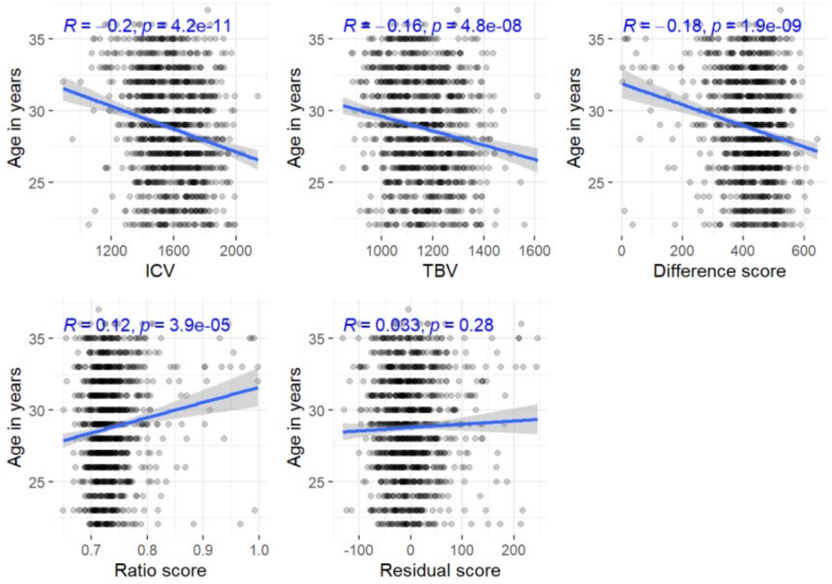

Methods Figure 5. Age correlations with ICV, TBV and the three atrophy scores including full HCP sample as downloaded.

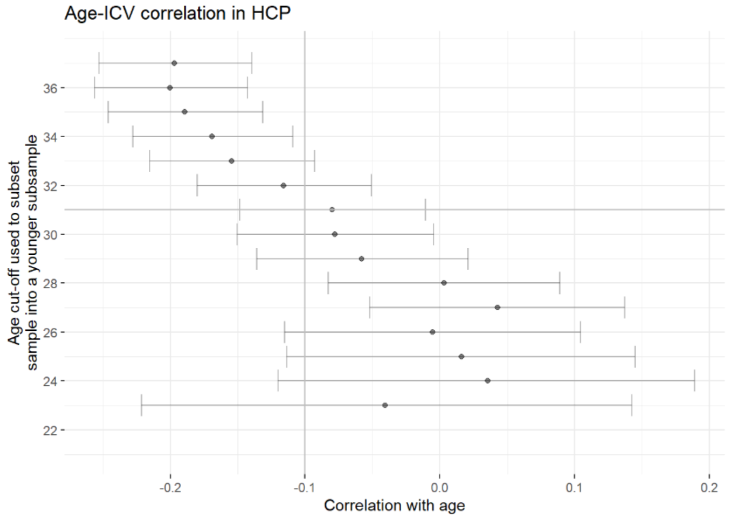

Methods Figure 6. Age ICV correlations in the HCP. *Y*-axis indicates the maximum age of each considered subsample

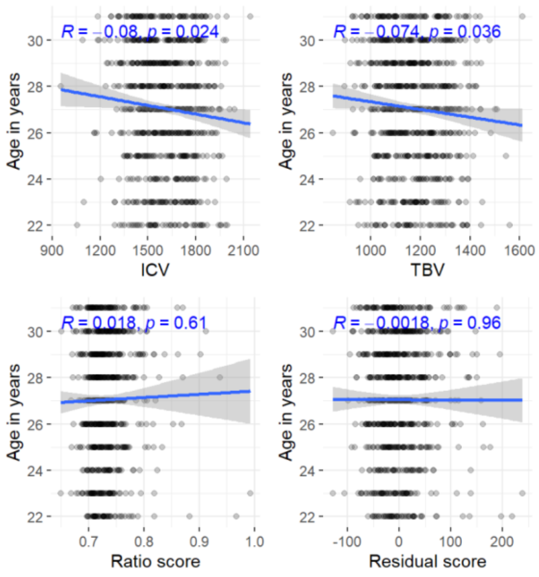

Methods Figure 7. Age correlations with ICV, TBV and the three atrophy scores including sample of HCP participants below the age of 31 years

An anonymous reviewer brought to our attention that the older females in HCP have unusually small ICVs. *Methods Figure 8* below (left) shows that the unexpected association between age and ICV is only significant in females, but not in males. *Methods Figure 8* (right) also shows that this bias is not present when we analyse <31-year-olds only. 203 of the 303 HCP participants that were >31-year and were excluded following the description above were females.

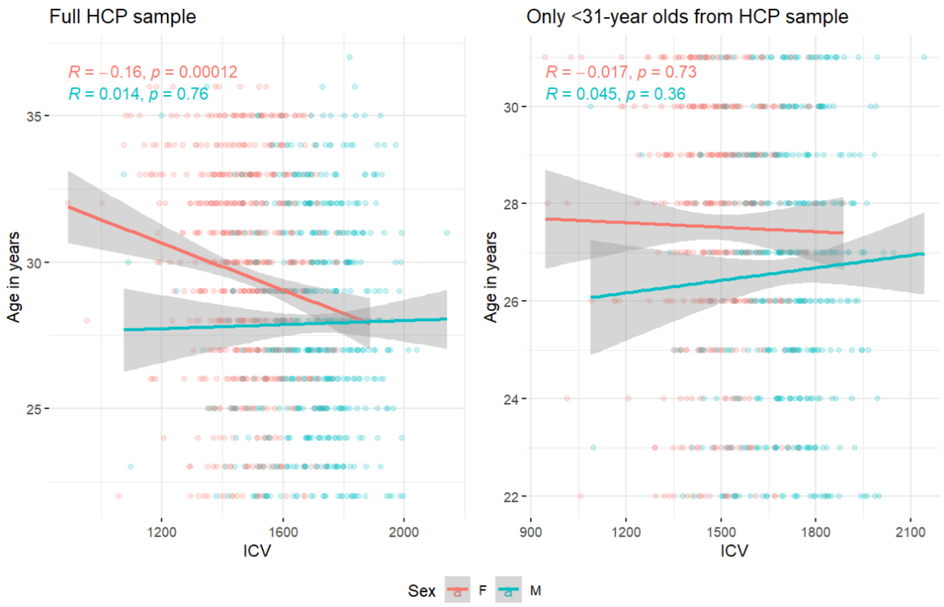

Methods Figure 8. Sex-split age correlations with ICV. Left: correlation in the full sample; right: correlation in <31-year-olds.

Pre-registered Aim 2; sample specificity of LBA norms and the role of ICV

Our pre-registered Aim 2 of this study had been designed to ensure fair comparisons between cross-sectionally- ‘estimated’ lifetime atrophy and longitudinally- ‘observed’ atrophic changes, where the former will have occurred across an entire *lifetime* and the latter can only span a time window between the two measured time points. We outlined the following analysis plan in order to approximately equate those two timelines: We planned to identify a pre-neurodegenerative subsample of the UKB that appeared to have no brain atrophy at the initial neuroimaging visit (according to one single MRI scan at initial neuroimaging visit). We had theorised that this sample may then demonstrate some loss in TBV between the first and the second neuroimaging visit, which would mean that timelines for lifetime atrophy and longitudinal atrophic changes should be more similar than if comparing estimated LBA with change in people who have already atrophied to some extent. As there is no objective criterion to determine whether a participant may fall into this pre-neurodegenerative category, we pre-registered to derive cut-off thresholds from independent, young cohorts (i.e., HCP and MRi-Share), where it is reasonable to assume that participants have not yet had meaningful neurodegeneration (mean age in HCP = 27 years, mean age in MRi-Share = 22 years).

Specifically, we assumed that young participants’ brains within 2 standard deviations in their difference, ratio, and residual scores to be representative of healthy-looking brains. We aimed to carry those raw 2SD cut-off values over to the UKB, to identify a subsample of participants with pre-neurodegenerative brains. Applying cut-offs derived from young cohorts to the older UKB cohort was intended to capture UKB participants whose brain atrophy scores implied similarly healthy-looking brains to those in the younger cohorts HCP and MRi-Share.

However, efforts to conduct this analysis failed because raw lifetime atrophy scores were highly sample-specific, and therefore not sensibly transferable between samples. This was likely driven by the fact that ICV measures were strongly discordant between samples [ICV means (SDs) in mm^3: MRi-Share = 1568.07 (140), HCP = 1606.3 (170), UKB = 1555.18 (150), LBC = 1396.53 (140)]. A cross-sample evaluation of raw atrophy scores would have misleadingly implied that older participants’ brains scanned in UKB and LBC looked just as healthy as young participants’ brains scanned in HCP and MRi-Share. That is, the raw difference between TBV and ICV was approximately the same for younger [Mean (SD) difference in mm^3: HCP = 432.49 (79.34); MRi-Share = 436.18 (48.29)] as for older samples [Mean (SD) in mm^3: UKB = 369.24 (69.44); LBC = 385.31 (86.88); $p = 1$]. This was the same for the raw ratio of ICV to TBV [Mean (SD) ratio: HCP = 0.73 (0.04); MRi-Share = 0.72 (0.02); UKB = 0.76 (0.03); LBC = 0.73 (0.05)] (*SFig.1-2*). This strong sample mismatch meant that our pre-registered Aim 2 analyses were not feasible or interpretable.

Moderation analysis

To better understand the relationship between LBA and longitudinal atrophic changes, we conducted a moderation analysis to investigate whether the association between observed atrophic changes (capturing change between time 1 and time 2) and LBA (at time 2), is moderated by the amount of LBA that occurred prior to a participants’ first MRI scan (at time 1). We hypothesised that the strongest correlation between those measures would occur when participants exhibit minimal atrophy at time 1, in which case atrophic changes and lifetime atrophy at time 2 would cover approximately the same timeline. Conversely, when the brain had already substantially atrophied prior to time 1, observed atrophic changes would likely capture fewer changes compared with lifetime measures because the latter should reflect the entire span of atrophic effects ever experienced – assuming that brain atrophy declines continually and linearly. This analysis was conceived and pre-registered as a sensitivity analysis to the failed Aim 2 analyses reported above. It had considerable issues of multicollinearity and the limited variance captured by longitudinally-estimated atrophy

prohibited the moderation analysis to produce interpretable results. The results were therefore not reported in the Supplementary Materials.

GWAS re-analysis to test whether results change when adjusting for fewer genetic PCs

It was brought to our attention after we had pre-registered our GWAS analyses, that 40 genetic PCs likely overcorrect GWAS results because some tend to capture complex linkage disequilibrium structure, which reduces power. We identified that our neuroimaging subset should best be controlled for 8 genetic PCs (as done in Privé et al., 2020), and re-calculated GWAS adjusting for 8 genetic PCs only. The GWAS results were highly similar when adjusting for 8 or for 40 genetic PCs. The comparison between the two is discussed in the main paper and in *Supplementary Data 9*.

Box S2. Lavaan syntax for GWAS-by-subtraction model

```
BaseICV=~NA*TBV + ICV
Atrophy=~NA*TBV
Atrophy ~~ 1*Atrophy
BaseICV ~~ 1*BaseICV
Atrophy~~0*BaseICV
ICV ~~ 0*TBV
ICV ~~ 0*ICV
TBV ~~ 0*TBV
Atrophy ~~ resid
BaseICV ~~ resid
```

Supplementary Results

Repeated LBA measures increase with chronological age in the LBC1936

Repeated MRI measures in the age-homogeneous LBC1936 showed that LBA increases with advancing age, even when MRI measures at each visit were processed independently of each other (i.e., here MRI data was processed with the FS cross-sectional rather than the longitudinal processing stream; *SFig.12*). According to all three computational methods, participants showed significantly more LBA at age 82 than at age 73 years [difference score: mean at age 70 (SD) = 375 (82) mm^3, mean at age 79 (SD) = 446 (102) mm^3; ratio score: mean at age 70 (SD) = 0.73 (0.04); mean at age 79 = 0.68 (0.05); residual score [1]: mean at age 70 (SD) = 40 (53); mean at age 79 = -37 (58); t-test *p*-values for the three scores < $5x10^{-14}$]. This section was conceived to compliment *Fig.3* in the main manuscript.

[1] In order to illustrate that the residual score indicated greater LBA with advancing age in this age-homogeneous sample, the residual score presented here was calculated across all measurements at 4 time points so that atrophy estimates were derived relative to the same average value. This was required to illustrate time-dependent increases, because all time points would have had the same average value had we derived the residual score for each individual time point independently, as was done for the residual score across the manuscript where only one time point was available. The LBC1936 data in this section was processed with the FS cross-sectional stream (as opposed to the longitudinally processed MRI data) where ICV is *not* held constant across visits, which should mimic a cross-cohort comparison, but likely makes the residual score noisier. It speaks to the validity of LBA that increases in the residual score remain prominent here.

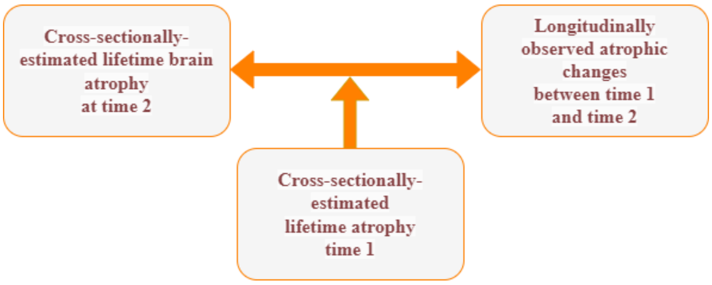

Illustration of the moderation analysis. Directed acyclic graph illustrating moderation analysis. This moderation suggests that the association between lifetime atrophy at time 2 and longitudinal atrophic changes between time 1 and time 2 is mediated by an individuals initial lifetime atrophy at time 1

Aim 3: Associations with ageing-related variables

Our pre-registered Aim 3 was designed to assess the external validity of cross-sectionally-estimated lifetime brain atrophy by quantifying the extent to which lifetime atrophy captures variance predictive of cognitive- and other ageing-related phenotypes (presented in Fig.2 in main manuscript for LBC1936 and SFig.7 for UKB). The pre-registered 3.1-3.4 analyses below aimed to further evaluate the nature of their relationship. We suspect that particularly the analyses in 3.2-3.3 suffer from variance inflation because we were including TBV, ICV or a variable derived from TBV and ICV in the same model. Results do not seem intuitive and it is likely that estimates were instable due to variance inflation, or other biases introduced by performing a regression that included both a derived measure (such as LBA $_{difference}$, LBA $_{ratio}$ or LBA $_{residual}$) and a baseline measure on which basis the first measure was derived (e.g., Bateson et al., 2019; Butler et al., 2021; Glymour et al., 2005). We have therefore not further interpreted the results displayed in the plots below. They are only included here for completeness as they had been pre-registered.

Aim 3.1: Amplifier effect

1. Health-related phenotypes ~ lifetime atrophy *time 2*
2. Health-related phenotypes ~ TBV *time 2*

Our lifetime brain atrophy definition using the residual score considers the residuals of TBV adjusted for ICV, and this analysis 3.1 demonstrated that this procedure resulted in an amplifier effect whereby the residuals explain more variance than the original/ unadjusted variable (i.e., TBV). We showed the existence of this amplifier effect in the LBC1936 whereby considering variance explained in an ageing-related trait we would sensibly expect to be associated with lifetime brain atrophy (e.g., visual rating scales, cognitive slope). We did not see evidence of an amplifier effect when considering variance in another trait that we would not expect to be associated with lifetime brain atrophy (e.g., cognitive intercept).

To illustrate the amplifier effect, we contrasted estimates of variances explained (R^2) from two models: the first predicting health outcomes with the residuals of TBV adjusted for ICV, and the second predicting the same health outcomes with TBV alone. Those residuals were obtained from either a difference, ratio, or residual method. R^2 estimates are plotted in Figure below.

Three overarching results from the LBC1936 are discussed below by focusing on lifetime brain atrophy scores obtained from the residual method, because it is the one score (out of the three computational methods) that is fully uncorrelated with ICV (i.e., baseline differences in head size). First, lifetime atrophy (R^2 = 11-20%), but not TBV alone (R^2 = 0.01-0.35%) are associated with visually rated atrophy, confirming that the residuals indeed carry information beyond simple baseline differences in TBV, that is relevant to clinically measured brain atrophy. Second, lifetime atrophy calculated with the residual method explained more than double the variance in a slope of cognitive function (R^2 = 13%) than can be explained with TBV alone (R^2 = 5%). This illustrates that adjusting for ICV acts as an amplifier in predicting a variable that is expected to be associated with neurodegeneration, but less so with baseline differences in head size. It complements this observation that TBV alone explains more variance in baseline cognitive ability (R^2 = 12%) than lifetime atrophy (R^2 = 0.02-8%), suggesting that atrophy scores indeed index variance related to change, and less so baseline differences in cognitive function. With respect to other traits for which we had longitudinal measures available and modelled intercepts and slopes, at least numerically it appears that estimated

atrophy tends to explain more variance in slopes (i.e., change), and TBV alone explains more variance in intercepts (i.e., baseline).

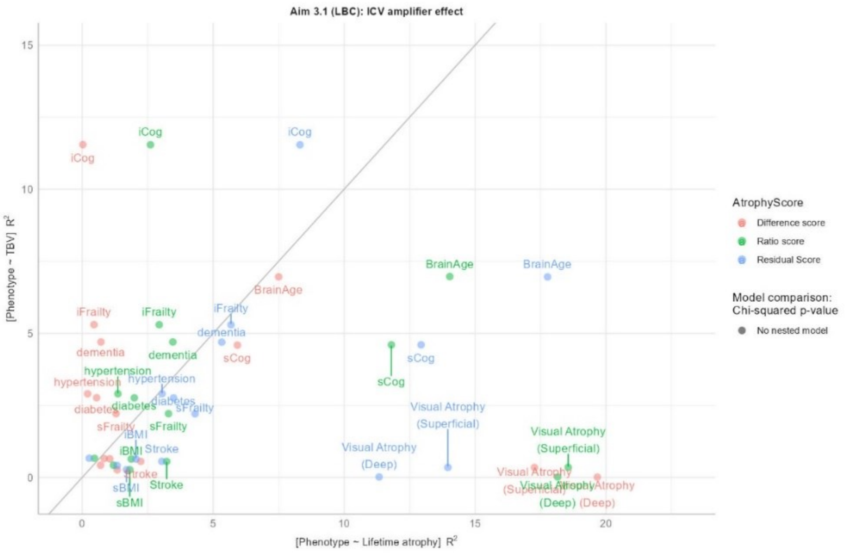

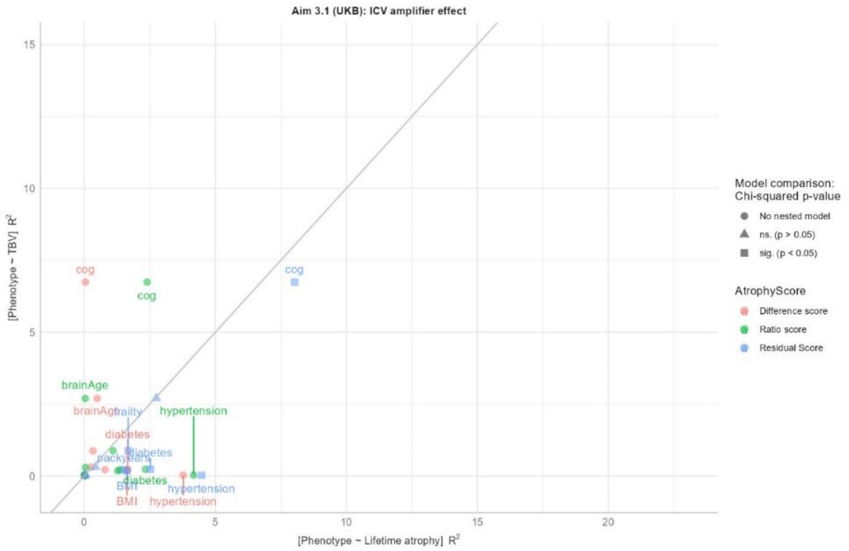

Linear regression x~y:

- UK Biobank: intercept = 1.17 (p = 0.003), slope = 0.29 (p = 0.078)
- LBC1936: intercept = 5.31 ($p = 9.25 \times 10^{-5}$), slope = 0.01 (p = 0.95)

Aim 3.2: Does lifetime atrophy (LBA) explain variance above and beyond baseline variables TBV and ICV?

1. Health-related phenotypes ~ *ICV time 2* (or *TBV time* 2) + *cross-sectional atrophy time 2*
2. Health-related phenotypes ~ *ICV time 2* (or *TBV time* 2)

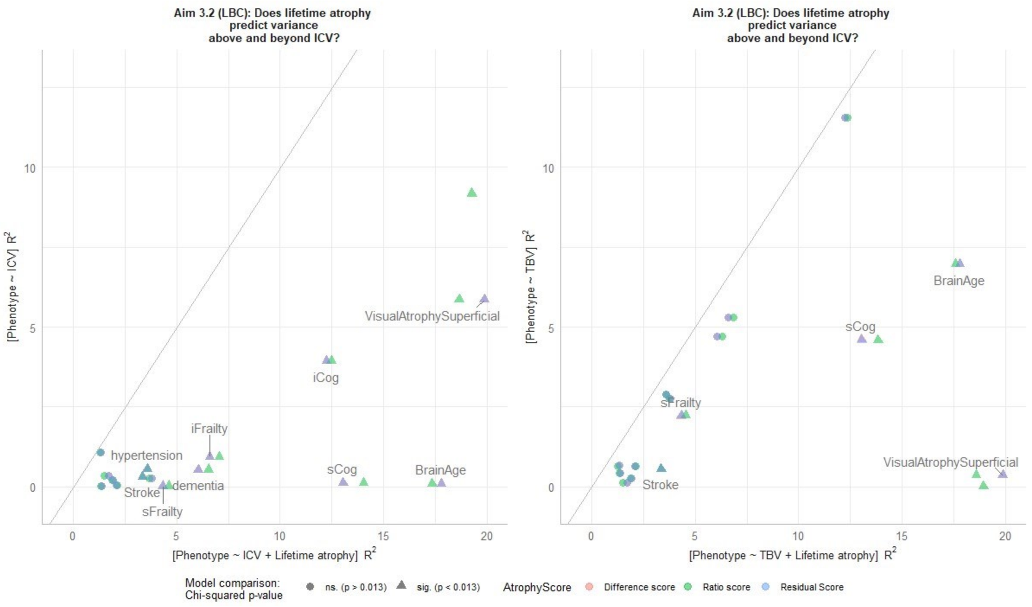

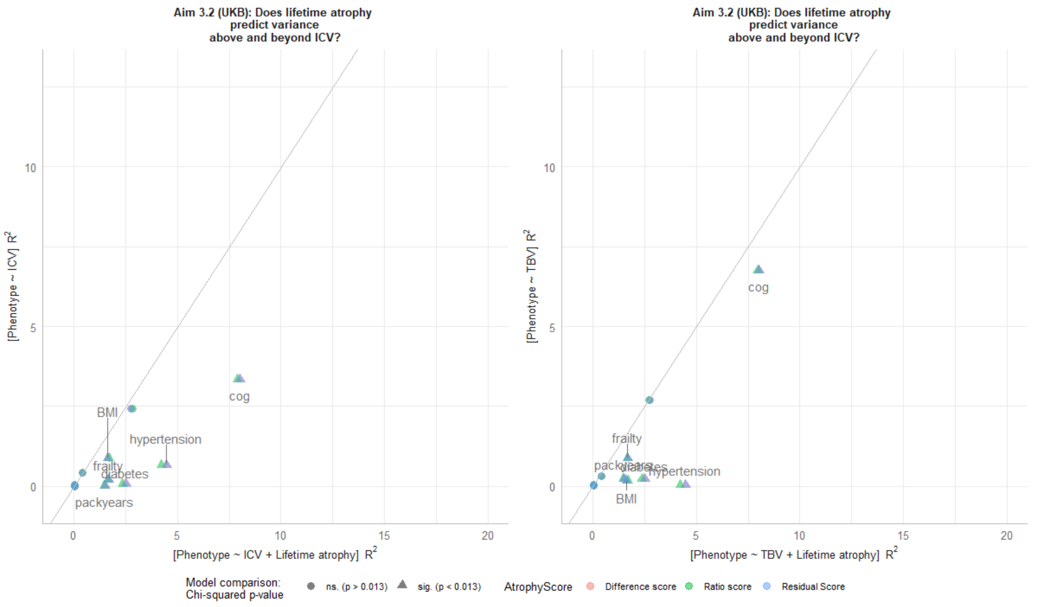

Linear regression x~y:

- UK Biobank (TBV analysis): intercept = 1.23 (p = 0.0001), slope = 0.94 ($p = 1.7\text{x}10^{-8}$)
- UK Biobank (ICV analysis): intercept = 0.96 (p = 0.006), slope = 1.66 ($p = 1.34\text{x}10^{-7}$)

- LBC1936 (TBV analysis): intercept = 5.55 (p = 4.14x10^{-5}), slope = 0.69 (p = 0.024)
- LBC1936 (ICV analysis): intercept = 4.73 (p = 2.47x10^{-7}), slope = 1.87 (p = 9.43x10^{-9})

Aim 3.3: Does lifetime brain atrophy explain health phenotype-associated variance above and beyond the variance explained by estimated atrophy?

1. Health-related phenotype ~ (*cross-sectionally) estimated atrophy time 2* + (*longitudinally) observed atrophy*
2. Health-related phenotype ~ (*cross-sectionally) estimated atrophy time 2*

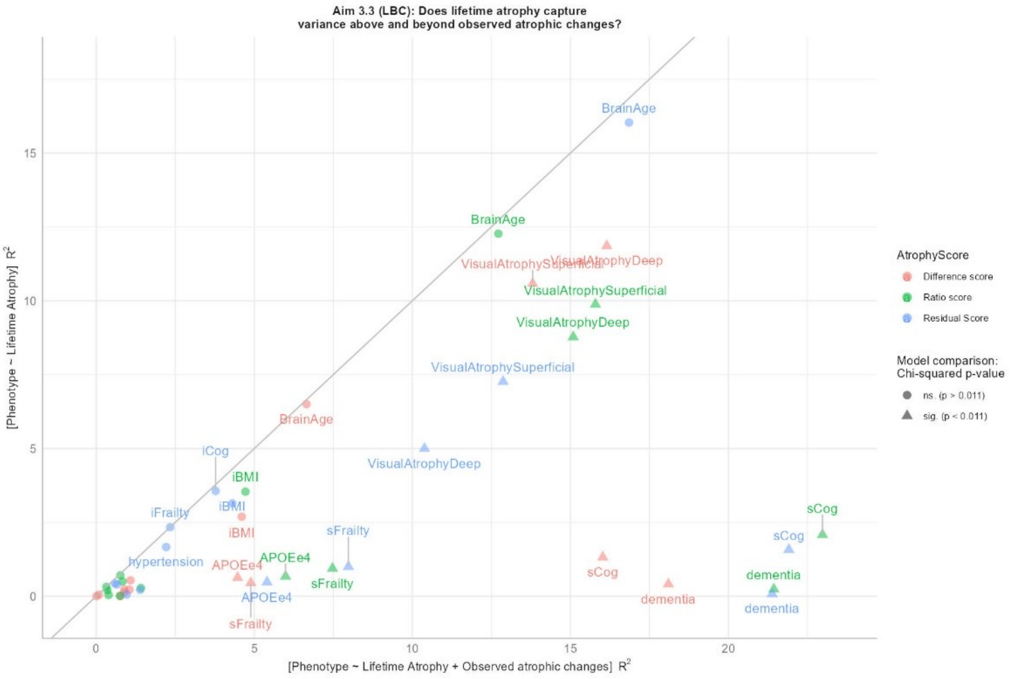

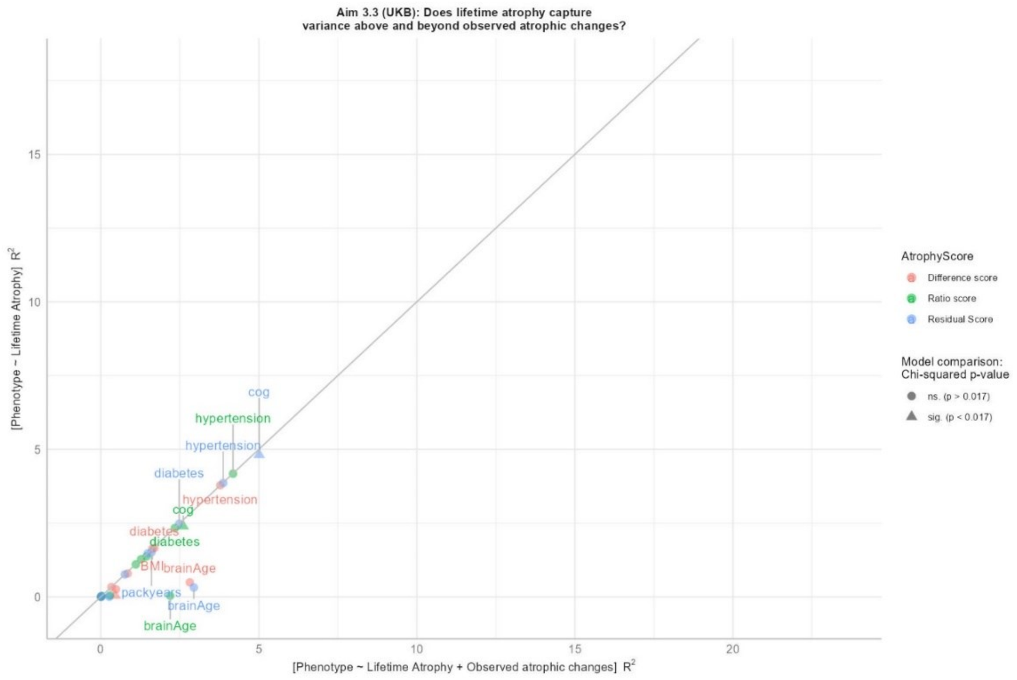

Linear regression x~y:

- UK Biobank: intercept = 0.45 (p = 0.0139), slope = 0.88 ($p = 3.04 \times 10^{-10}$)
- LBC1936: intercept = 4.62 (p = 0.0003), slope = 0.89 (p = 0.0007)

Aim 3.4: General trends of associations across aims 3.1-3.3

Simply from looking at the plots above, there do not seem to be obvious overarching trends. Linear regressions reporting intercepts and slopes do not deliver strong evidence for consistent trends across UKB and LBC1936. The regression results may be summarised as follows:

- Aim 3.1 (amplifier effect): Both samples yielded non-significant slopes between predicted R^2 by TBV alone vs. R^2 by LBA. Some outcome traits were more strongly associated with TBV and others were more strongly associated with LBA. For example, in LBC1936, the amplifier effect seemed to affect traits like cognitive slopes and visual atrophy scales but not cognitive intercepts.
- Aim 3.2: In general, LBA added explanatory variance above and beyond variance explained by baseline levels of TBV or ICV, which was indicated by a significant overarching slope in LBC1936 and UKB.
- Aim 3.3: Both cohorts produce different results with regard to whether longitudinally-observed atrophic changes add variance above and beyond variance explained by LBA alone. In UKB, observed atrophic changes do not tend to add explanatory variance above and beyond LBA (slope near 1, and intercept near 0), which may be driven by the limited variance captured in this longitudinal measure (*SFig.8*). In the LBC1936, observed atrophic changes tend to add explanatory variance above and beyond LBA suggesting LBA and observed atrophy changes capture meaningful variance in their own right. This is consistent with the interpretation in the main manuscript that LBA seems to capture longer-term changes

of ageing that are not specifically disease-related and that longitudinally-observed atrophic changes capture more short-term brain shrinkage that is associated with dementia.

SNP-by-age interaction effects on LBA

Given the strong association between LBA and age, we aimed to explore how SNP effects might be moderated by age through a SNP-by-age interaction analysis. This analysis helps clarify whether the genetic associations we observe are consistently significant across different age groups or if their strength and significance change with advancing age. Interaction tests were calculated in REGENIE (Mbatchou et al., 2021) where we added the entry-wise product of SNP and age to the GWAS model. From this model we extracted the marginal SNP effects (REGENIE label: ADD-INT_SNP) and the SNP-by-age interaction effects (REGENIE label: ADD-INT_SNPxAge) for SNPs that were of primary interest under a polygenic model (minor allele frequency; MAF > 0.01, INFO > 0.9, biallelic). For $LBA_{residual}$, the interaction model produced a very similar-appearing Manhattan plot as the original GWAS model (*SFig.20*). Marginal GWAS SNP effects on LBA remained very similar compared to SNP effects obtained from a model excluding the SNP-by-age interaction term: the main SNP betas from the original GWAS vs. the marginal SNP effects from the interaction GWAS were perfectly correlated (intercept = 0, slope = 1; *SFig.24*). Zero SNPs showed significant SNP-by-age interaction effects ($p < 5 \times 10^{-8}$; *SFig.29*), which included both *APOE* SNPs rs7412 and rs429358 ($p_{rs7412} = 0.195$; $p_{rs429358} = 0.660$). This could be due to the typical lack of power to detect modest SNP-by-age interaction effect sizes. Those SNP-by-age interaction trends were very similar for both the difference and ratio scores, as well as TBV (*SFig.20-32*). The lack of SNP-by-age interaction effects may suggest that our measure of brain change (i.e., LBA) is largely independent of age meaning that age-associated variance has been absorbed and is represented by the LBA measure.

Additionally, we calculated the LBA variance explained by SNP-by-age interactions using GCTA (Yang et al., 2011) to obtain an estimate independent of individual SNP effects (*Supplementary Data 8*). Although statistically non-significant in $N = 38{,}624$, point estimates for the gene-by-age interaction insinuated a sizable contribution to phenotypic variance (34%; $SE = 0.83$), but the large standard errors underlined the lack of power for GxE interaction models. Their reliable detection will require much larger samples.

GWAS re-analysis adjusting for 8 genetic PCs only (instead of 40 genetic PCs as pre-registered)

We analysed the UKB neuroimaging subset following the tool described in (Privé et al., 2020). The plots below illustrate that the first 8 genetic PCs capture genome-wide-associated population structure. Genetic PCs 9-40, by contrast, capture complex LD structure. This is illustrated by plotting LD loadings against one another – only PCs 1-8 seem to capture systematic variance rather than random noise. Genetic PCs 9-40 visibly capture towers of LD structure as is illustrated in the Manhattan-like plots that show PC loadings for each hex-binned genetic variant on the genome. The scree plot indicates a relatively steeper increase in eigenvalue between genetic PC 8 and 9, supporting evidence that the first 8 PCs capture most of the variability across variants.

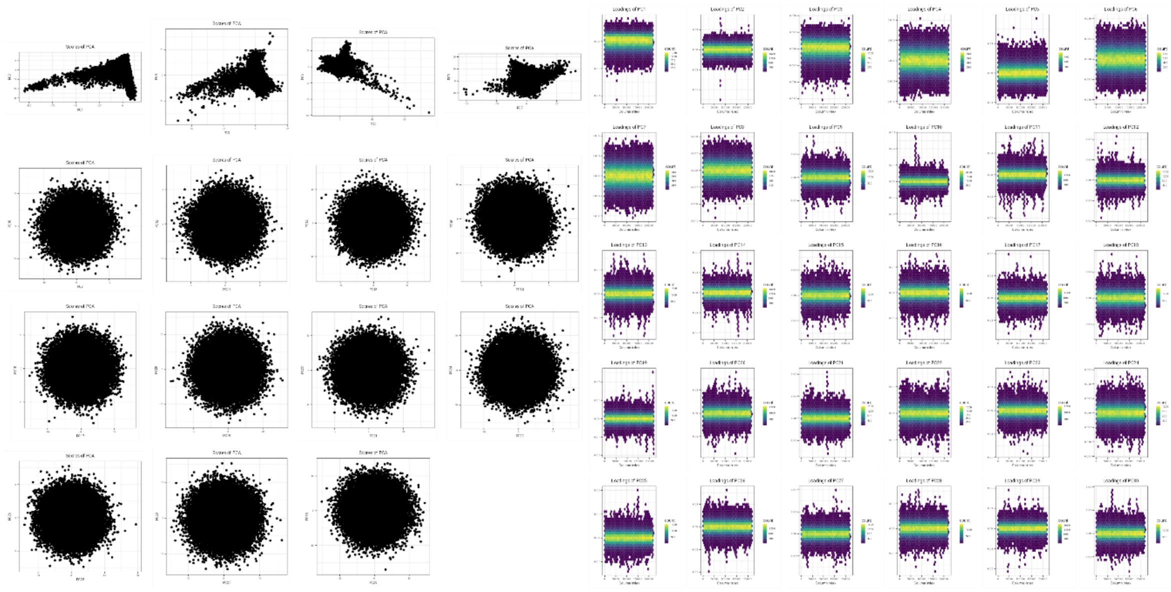

Left: PC loadings from different PCs plotted against one another; Right: PC loadings plotted onto their genomic location (Manhattan-like plot)

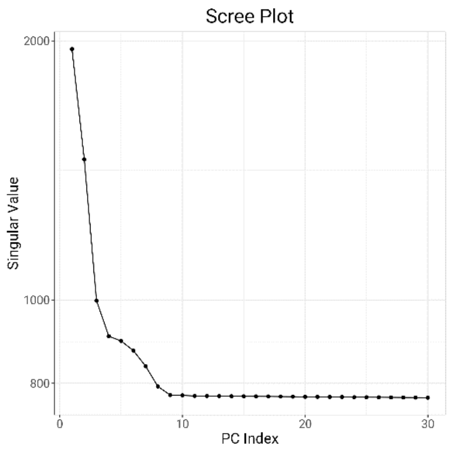

Scree plot from the genetic Principal Component Analysis of the neuroimaging subset

Based on this empirical evidence obtained from Privé et al. (2020)'s tool, we re-calculated GWAS reported in the main manuscript, to test whether including fewer PCs substantially altered the GWAS results. Summary statistics reported in *Supplementary Data 9* were identical for LBA $_{residual}$. LBA $_{residual}$ adjusted for 40 genetic PCs was genetically correlated with LBA $_{residual}$ adjusted for 8 PCs at $r_g = 0.998$ (SE = 0.07). LBA $_{ratio}$ adjusted for 40 genetic PCs was genetically correlated with LBA $_{ratio}$ adjusted for 8 PCs at $r_g = 0.998$ (SE = 0.07). LBA $_{difference}$ adjusted for 40 genetic PCs was genetically correlated with LBA $_{difference}$ adjusted for 8 PCs at $r_g = 0.998$ (SE = 0.07). FUMA indicated that LBA difference and LBA ratio captured a larger count of independent significant GWAS hits and more GWAS loci in the GWAS adjusting for 8 instead of 40 genetic PCs. Empirical investigations should test whether this complex LD structure weakly that we seem to overcorrect by including 40 genetic PCs tags pre-existing anthropometric information, like height, and skull size, which could explain the impact PC adjustment had on LBA $_{difference}$ and LBA $_{ratio}$, but not on LBA $_{residual}$.

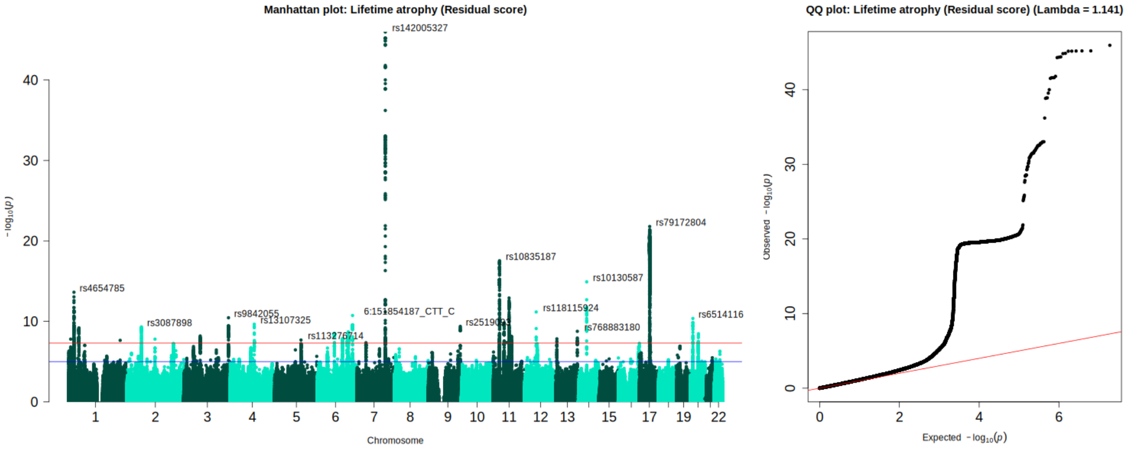

Residual score: Manhattan (left) and quantile-quantile plot (right) for lifetime brain atrophy indicating top GWAS hits

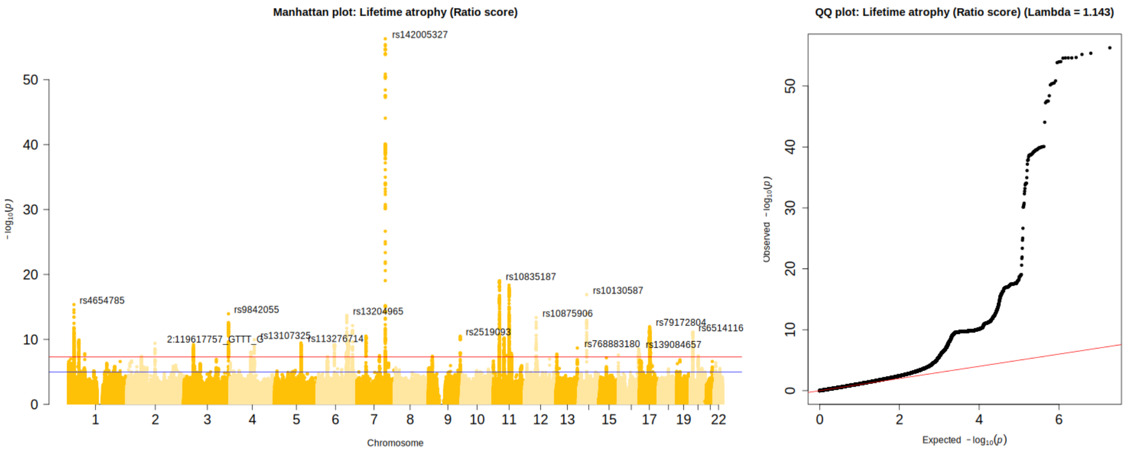

Ratio score: Manhattan (left) and quantile-quantile plot (right) for lifetime brain atrophy indicating top GWAS hits

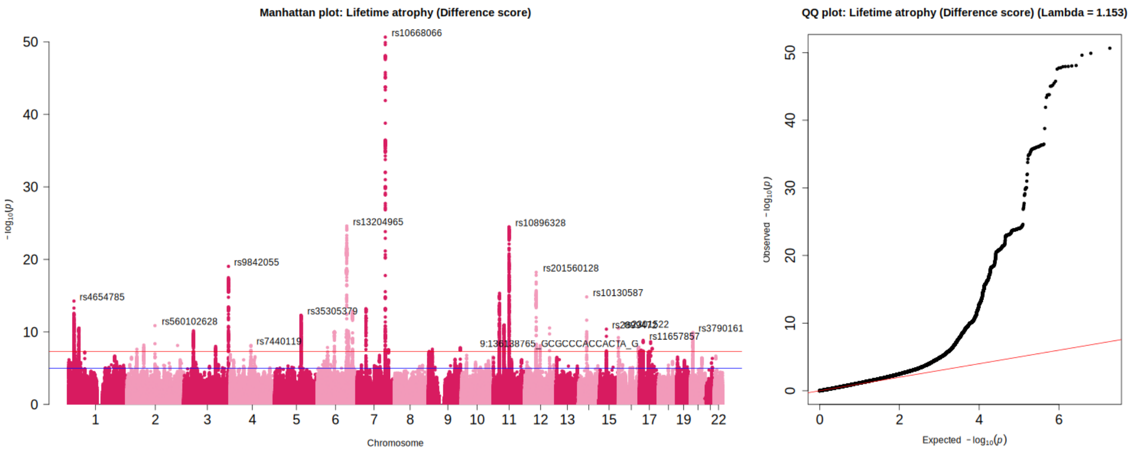

Difference score: Manhattan (left) and quantile-quantile plot (right) for lifetime brain atrophy indicating top GWAS hits

References

Archie, J. P., Jr. (1981). Mathematic coupling of data: a common source of error. *Ann Surg*, *193*(3), 296-303. https://doi.org/10.1097/00000658-198103000-00008

Bateson, M., Eisenberg, D. T., & Nettle, D. (2019). Controlling for baseline telomere length biases estimates of the rate of telomere attrition. *Royal Society open science*, *6*(10), 190937. https://doi.org/doi.org/10.1098/rsos.190937

Bu, N., Khlif, M. S., Lemmens, R., Wouters, A., Fiebach, J. B., Chamorro, A., Ringelstein, E. B., Norrving, B., Laage, R., Grond, M., Wilms, G., Brodtmann, A., & Thijs, V. (2021). Imaging Markers of Brain Frailty and Outcome in Patients With Acute Ischemic Stroke. *Stroke*, *52*(3), 1004-1011. https://doi.org/doi:10.1161/STROKEAHA.120.029841

Butler, E. R., Chen, A., Ramadan, R., Le, T. T., Ruparel, K., Moore, T. M., Satterthwaite, T. D., Zhang, F., Shou, H., Gur, R. C., Nichols, T. E., & Shinohara, R. T. (2021). Pitfalls in brain age analyses. *Human Brain Mapping*, *42*(13), 4092-4101. https://doi.org/https://doi.org/10.1002/hbm.25533

Caspi, Y., Brouwer, R. M., Schnack, H. G., van de Nieuwenhuijzen, M. E., Cahn, W., Kahn, R. S., Niessen, W. J., van der Lugt, A., & Pol, H. H. (2020). Changes in the intracranial volume from early adulthood to the sixth decade of life: A longitudinal study. *NeuroImage*, *220*, 116842. https://doi.org/https://doi.org/10.1016/j.neuroimage.2020.116842

Clifton, L., & Clifton, D. A. (2019). The correlation between baseline score and post-intervention score, and its implications for statistical analysis. *Trials*, *20*(1), 43. https://doi.org/10.1186/s13063-018-3108-3

Cole, J. H., Ritchie, S. J., Bastin, M. E., Valdés Hernández, M. C., Muñoz Maniega, S., Royle, N., Corley, J., Pattie, A., Harris, S. E., Zhang, Q., Wray, N. R., Redmond, P., Marioni, R. E., Starr, J. M., Cox, S. R., Wardlaw, J. M., Sharp, D. J., & Deary, I. J. (2018). Brain age predicts mortality. *Molecular Psychiatry*, *23*(5), 1385-1392. https://doi.org/10.1038/mp.2017.62

Cox, S. R., Lyall, D. M., Ritchie, S. J., Bastin, M. E., Harris, M. A., Buchanan, C. R., Fawns-Ritchie, C., Barbu, M. C., de Nooij, L., Reus, L. M., Alloza, C., Shen, X., Neilson, E., Alderson, H. L., Hunter, S., Liewald, D. C., Whalley, H. C., McIntosh, A. M., Lawrie, S. M., . . . Deary, I. J. (2019). Associations between vascular risk factors and brain MRI indices in UK Biobank. *European Heart Journal*, *40*(28), 2290-2300. https://doi.org/10.1093/eurheartj/ehz100

Cronbach, L. J., & Furby, L. (1970). How we should measure "change": Or should we? *Psychological Bulletin*, *74*(1), 68-80. https://doi.org/10.1037/h0029382

Farrell, C., Chappell, F., Armitage, P. A., Keston, P., MacLullich, A., Shenkin, S., & Wardlaw, J. M. (2009). Development and initial testing of normal reference MR images for the brain at ages 65–70 and 75–80 years. *European Radiology*, *19*(1), 177-183. https://doi.org/10.1007/s00330-008-1119-2

Fried, L. P., Tangen, C. M., Walston, J., Newman, A. B., Hirsch, C., Gottdiener, J., Seeman, T., Tracy, R., Kop, W. J., Burke, G., & McBurnie, M. A. (2001). Frailty in Older Adults: Evidence for a Phenotype. *The Journals of Gerontology: Series A*, *56*(3), M146-M157. https://doi.org/10.1093/gerona/56.3.M146

Fürtjes, A. E., Coleman, J. R. I., Tyrrell, J., Lewis, C. M., & Hagenaars, S. P. (2022). Associations and limited shared genetic aetiology between bipolar disorder and cardiometabolic traits in the UK Biobank. *Psychological Medicine*, *52*(16), 4039-4048. https://doi.org/10.1017/S0033291721000945

Glymour, M. M., Weuve, J., Berkman, L. F., Kawachi, I., & Robins, J. M. (2005). When Is Baseline Adjustment Useful in Analyses of Change? An Example with Education and Cognitive Change. *American Journal of Epidemiology*, *162*(3), 267-278. https://doi.org/10.1093/aje/kwi187

Good, C. D., Scahill, R. I., Fox, N. C., Ashburner, J., Friston, K. J., Chan, D., Crum, W. R., Rossor, M. N., & Frackowiak, R. S. (2002). Automatic differentiation of anatomical patterns in the human brain: validation with studies of degenerative dementias. *NeuroImage*, *17*(1), 29-46. https://doi.org/10.1006/nimg.2002.1202

Jiang, R., Noble, S., Sui, J., Yoo, K., Rosenblatt, M., Horien, C., Qi, S., Liang, Q., Sun, H., Calhoun, V. D., & Scheinost, D. (2023). Associations of physical frailty with health outcomes and brain structure in 483 033 middle-aged and older adults: a population-based study from the UK Biobank. *The Lancet Digital Health*, *5*(6), e350-e359. https://doi.org/10.1016/S2589-7500(23)00043-2

Kilsdonk, I., Steenwijk, M., Pouwels, P., Zwanenburg, J., Visser, F., Luijten, P., Geurts, J., Barkhof, F., & Wattjes, M. (2015). Perivascular spaces in MS patients at 7 Tesla MRI: A marker of neurodegeneration? *Multiple Sclerosis Journal*, *21*(2), 155-162. https://doi.org/10.1177/1352458514540358

Koedam, E. L. G. E., Lehmann, M., van der Flier, W. M., Scheltens, P., Pijnenburg, Y. A. L., Fox, N., Barkhof, F., & Wattjes, M. P. (2011). Visual assessment of posterior atrophy development of a MRI rating scale. *European Radiology*, *21*(12), 2618-2625. https://doi.org/10.1007/s00330-011-2205-4

Kuo, C.-L., Pilling, L. C., Atkins, J. L., Kuchel, G. A., & Melzer, D. (2020). ApoE e2 and aging-related outcomes in 379,000 UK Biobank participants. *Aging (Albany NY)*, *12*(12), 12222. https://doi.org/https://doi.org/10.18632/aging.103405

Lord, F. M. (1956). The measurement of growth. *Educational and Psychological Measurement*(16), 421-437.

Ma, D., Popuri, K., Bhalla, M., Sangha, O., Lu, D., Cao, J., Jacova, C., Wang, L., Beg, M. F., & Initiative, A. s. D. N. (2019). Quantitative assessment of field strength, total intracranial volume, sex, and age effects on the goodness of harmonization for volumetric analysis on the ADNI database. *Human Brain Mapping*, *40*(5), 1507-1527. https://doi.org/https://doi.org/10.1002/hbm.24463

Mbatchou, J., Barnard, L., Backman, J., Marcketta, A., Kosmicki, J. A., Ziyatdinov, A., Benner, C., O'Dushlaine, C., Barber, M., Boutkov, B., Habegger, L., Ferreira, M., Baras, A., Reid, J., Abecasis, G., Maxwell, E., & Marchini, J. (2021). Computationally efficient whole-genome regression for quantitative and binary traits. *Nature Genetics*, *53*(7), 1097-1103. https://doi.org/10.1038/s41588-021-00870-7

Muñoz Maniega, S., Meijboom, R., Chappell, F. M., Valdés Hernández, M. d. C., Starr, J. M., Bastin, M. E., Deary, I. J., & Wardlaw, J. M. (2019). Spatial Gradient of Microstructural Changes in Normal-Appearing White Matter in Tracts Affected by White Matter Hyperintensities in Older Age [Original Research]. *Frontiers in Neurology*, *10*. https://doi.org/10.3389/fneur.2019.00784

Nerland, S., Stokkan, T. S., Jørgensen, K. N., Wortinger, L. A., Richard, G., Beck, D., van der Meer, D., Westlye, L. T., Andreassen, O. A., Agartz, I., & Barth, C. (2022). A comparison of intracranial volume estimation methods and their cross-sectional and longitudinal associations with age. *Human Brain Mapping*, *43*(15), 4620-4639. https://doi.org/https://doi.org/10.1002/hbm.25978

Pasquier, F., Leys, D., Weerts, J. G. E., Mounier-Vehier, F., Barkhof, F., & Scheltens, P. (1996). Inter- and Intraobserver Reproducibility of Cerebral Atrophy Assessment on MRI Scans with Hemispheric Infarcts. *European Neurology*, *36*(5), 268-272. https://doi.org/10.1159/000117270

Privé, F., Luu, K., Blum, M. G. B., McGrath, J. J., & Vilhjálmsson, B. J. (2020). Efficient toolkit implementing best practices for principal component analysis of population genetic data. *Bioinformatics*, *36*(16), 4449-4457. https://doi.org/10.1093/bioinformatics/btaa520

Rogosa, D. R., & Willett, J. B. (1983). Demonstrating the Reliability of the Difference Score in the Measurement of Change. *Journal of Educational Measurement*, *20*(4), 335-343. http://www.jstor.org/stable/1434950

Rosseel, Y. (2012). lavaan: An R package for structural equation modeling. *Journal of statistical software*, *48*, 1-36. https://doi.org/10.18637/jss.v048.i02

Royle, N. A., Hernández, M. C., Maniega, S. M., Arabisala, B. S., Bastin, M. E., Deary, I. J., & Wardlaw, J. M. (2013). Influence of thickening of the inner skull table on intracranial volume measurement in older people. *Magn Reson Imaging*, *31*(6), 918-922. https://doi.org/10.1016/j.mri.2013.01.012

Rudick, R. A., Fisher, E., Lee, J.-C., Simon, J., Jacobs, L., & Group, t. M. S. C. R. (1999). Use of the brain parenchymal fraction to measure whole brain atrophy in relapsing-remitting MS. *Neurology*, *53*(8), 1698-1698. https://doi.org/10.1212/wnl.53.8.1698

Scheltens, P., Launer, L. J., Barkhof, F., Weinstein, H. C., & van Gool, W. A. (1995). Visual assessment of medial temporal lobe atrophy on magnetic resonance imaging: Interobserver reliability. *Journal of Neurology*, *242*(9), 557-560. https://doi.org/10.1007/BF00868807

Scheltens, P., Leys, D., Barkhof, F., Huglo, D., Weinstein, H. C., Vermersch, P., Kuiper, M., Steinling, M., Wolters, E. C., & Valk, J. (1992). Atrophy of medial temporal lobes on MRI in "probable" Alzheimer's disease and normal ageing: diagnostic value and neuropsychological correlates. *Journal of Neurology, Neurosurgery & Psychiatry*, *55*(10), 967-972. https://doi.org/10.1136/jnnp.55.10.967

Velickaite, V., Ferreira, D., Lind, L., Ahlström, H., Kilander, L., Westman, E., & Larsson, E. M. (2020). Visual rating versus volumetry of regional brain atrophy and longitudinal changes over a 5-year period in an elderly population. *Brain Behav*, *10*(7), e01662. https://doi.org/10.1002/brb3.1662

Welstead, M., Muniz-Terrera, G., Russ, T. C., Corley, J., Taylor, A. M., Gale, C. R., & Luciano, M. (2020). Inflammation as a risk factor for the development of frailty in the Lothian Birth Cohort 1936. *Experimental Gerontology*, *139*, 111055. https://doi.org/https://doi.org/10.1016/j.exger.2020.111055

Yang, J., Lee, S. H., Goddard, M. E., & Visscher, P. M. (2011). GCTA: a tool for genome-wide complex trait analysis. *The American Journal of Human Genetics*, *88*(1), 76-82. https://doi.org/10.1016/j.ajhg.2010.11.011

Supplementary plots

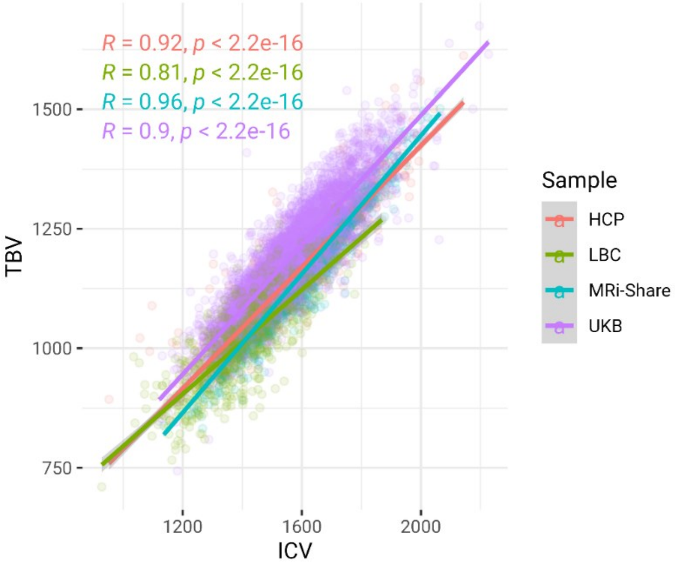

Fig.S1. Relationship between TBV (total brain volume) and ICV (intracranial volume) in all four considered cohorts. All three cohorts produced widely differing intercept and slope estimates: HCP (intercept = 161.77, slope = 0.63, *N* = 800), Share (intercept = -4.42, slope = 0.72, *N* = 1,831), UKB (intercept = 132.56, slope = 0.68, *N* = 4,674), LBC1931 (intercept = 249.58, slope = 0.54, *N* = 634)

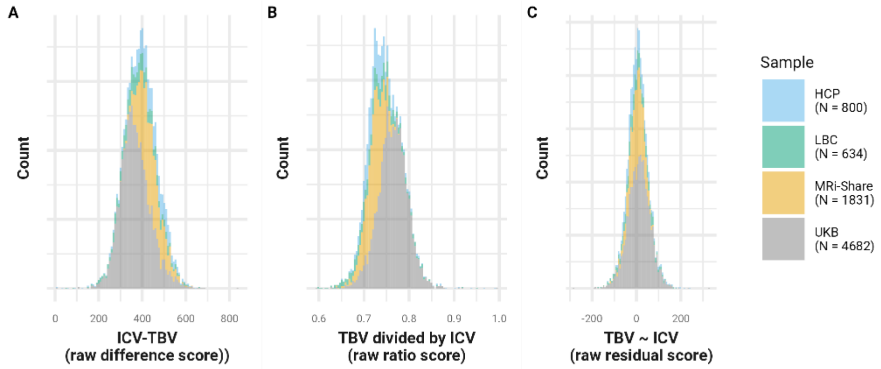

Fig.S2. Distribution of lifetime brain atrophy scores in considered samples. (A) Difference score, (B) ratio score, (C) residual score. HCP: Human Connectome Project; LBC: Lothian Birth Cohort 1938; UKB: UK Biobank. Regardless of their average sample age, those distributions look very similar, misleadingly indicating that brains scanned in the LBC and UKB cohorts appear just as healthy as those in the MRi-Share and HCP cohorts.

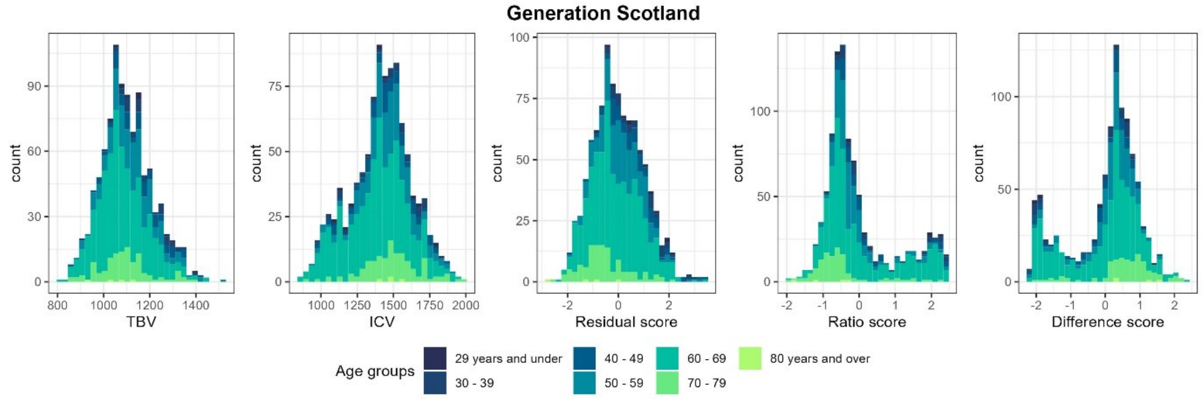
Generation Scotland
count
TBV
ICV
Residual score
Ratio score
Difference score
Age groups
29 years and under
30 - 39
40 - 49
50 - 59
60 - 69
70 - 79
80 years and over

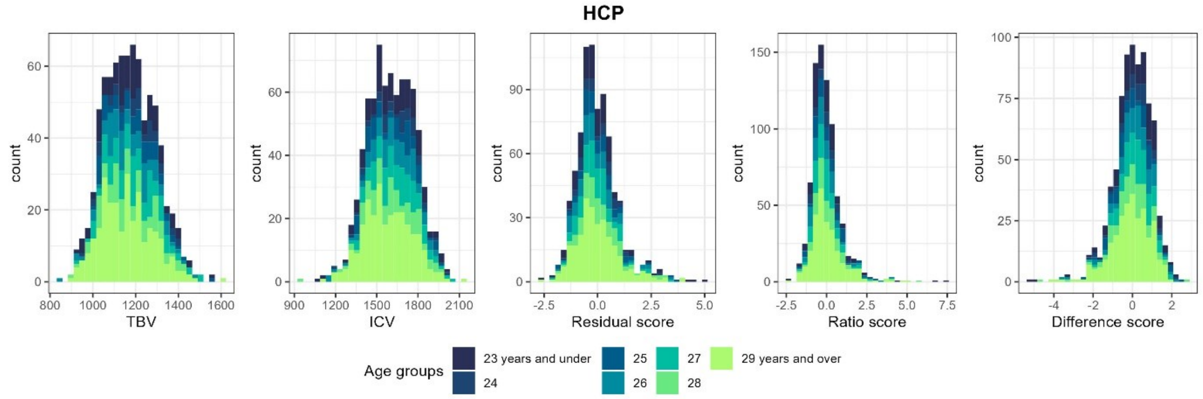
HCP
count
TBV
ICV
Residual score
Ratio score
Difference score
Age groups
23 years and under
24
25
26
27
28
29 years and over

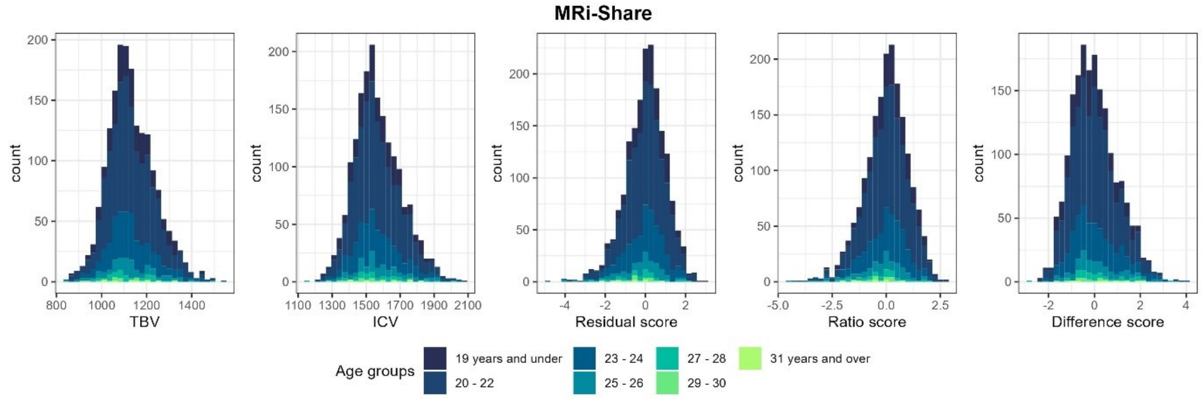
MRi-Share
count
TBV
ICV
Residual score
Ratio score
Difference score
Age groups
19 years and under
20 - 22
23 - 24
25 - 26
27 - 28
29 - 30
31 years and over

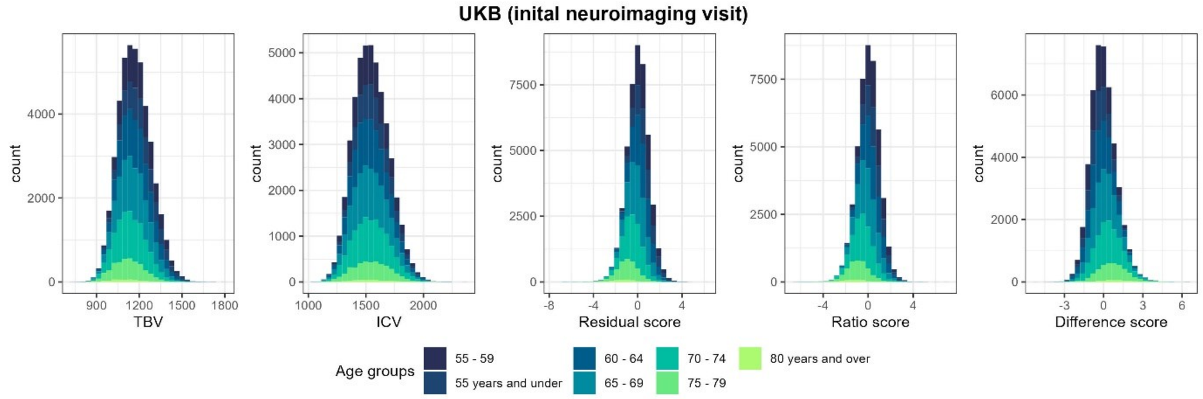
UKB (inital neuroimaging visit)
count
TBV
ICV
Residual score
Ratio score
Difference score
Age groups
55 - 59
55 years and under
60 - 64
65 - 69
70 - 74
75 - 79
80 years and over

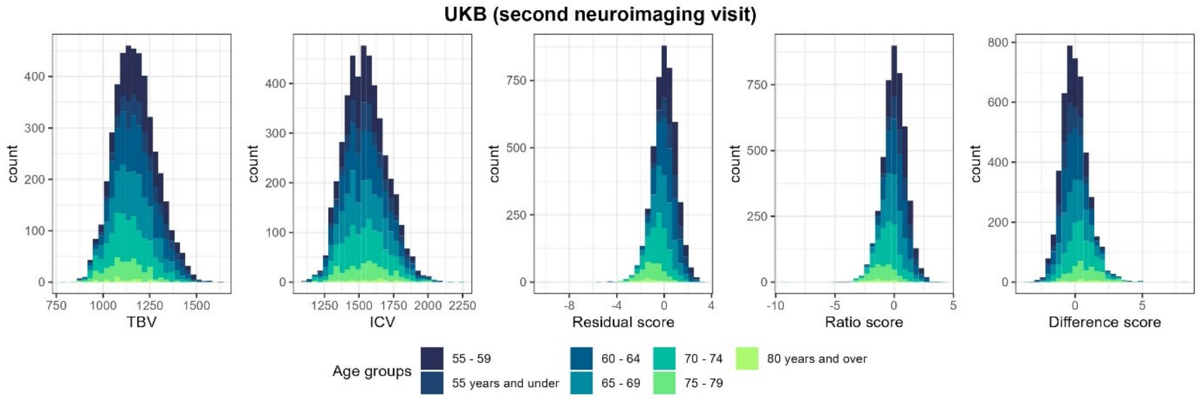

Fig.S3. Distributions of TBV, ICV, and lifetime brain atrophy estimated with the residual, ratio, and difference method. Histograms are coloured by age groups. Sample sizes (*N*): Generation Scotland = 987, HCP = 900, MRi-Share = 1,831, UKB initial visit = 43,110, UKB second visit = 4674

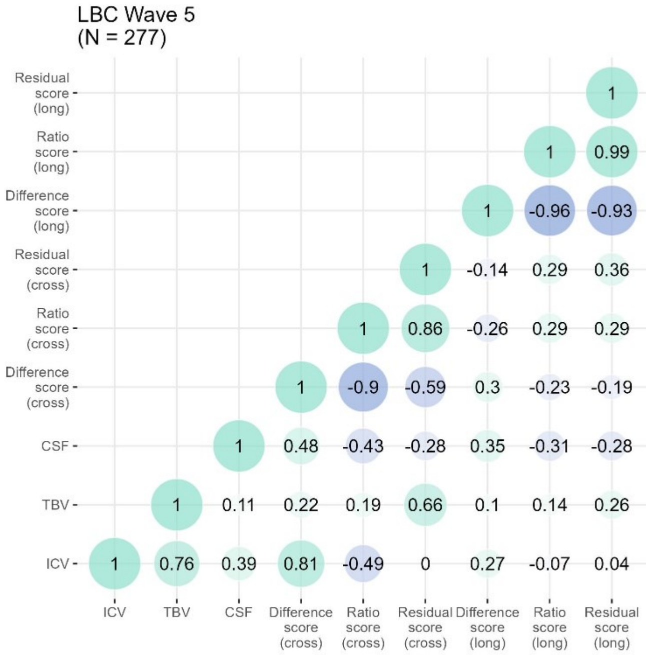

Fig.S4. Pearson’s correlations in the LBC1936 cohort (wave 5) among TBV, ICV, CSF, lifetime atrophy scores inferred with three computational methods (‘cross’), and longitudinally-observed atrophic changes inferred with three computational methods (‘long’). All measures were automatically extracted with FreeSurfer.

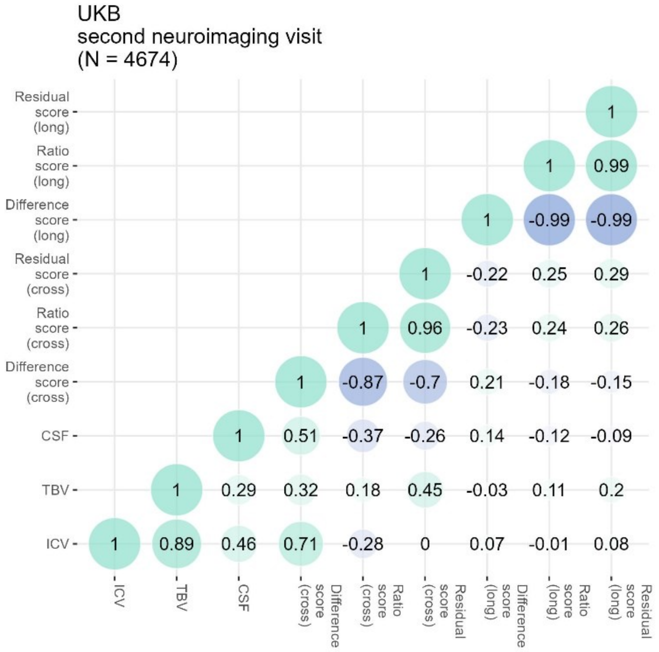

Fig.S5. Pearson's correlations in the UKB cohort among TBV, ICV, CSF, lifetime atrophy scores inferred with three computational methods ('cross'), and longitudinally-observed atrophic changes inferred with three computational methods ('long'). All measures were automatically extracted with FreeSurfer.

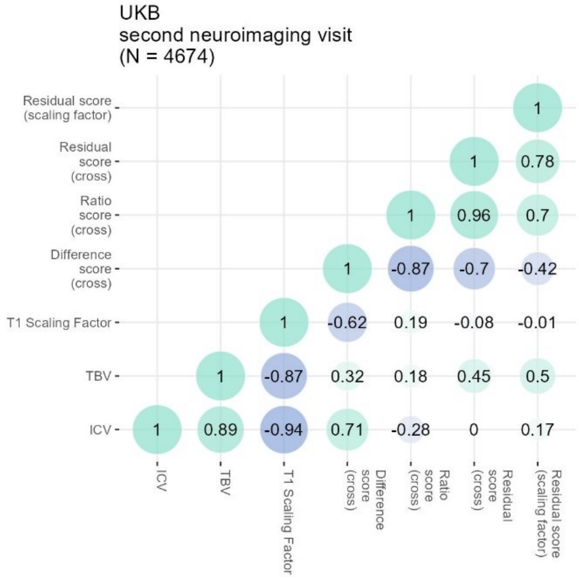

Fig.S6. Pearson's correlations in the UKB cohort among TBV, ICV, T1 Scaling Factor, and lifetime atrophy scores inferred with three computational methods ('cross'), as well as lifetime atrophy inferred with a residual score using the T1 scaling factor instead of ICV ('scaling factor')

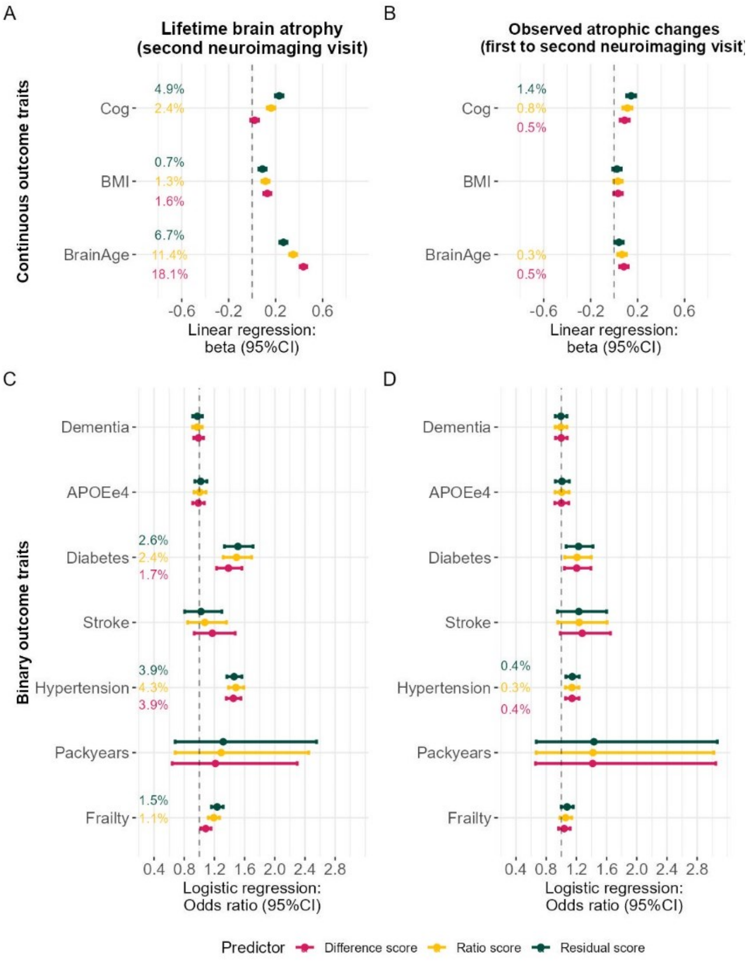

Fig.S7. Associations with health-related phenotypes in UKB for 'estimated' LBA and longitudinally-'observed' atrophic changes (N = 4,674). (A) Associations between LBA and continuous traits, (B) Associations between longitudinally-observed atrophic changes and continuous traits, (C) Associations between LBA and binary traits, (D) Associations between longitudinally-observed atrophic changes and binary traits. Abbreviations: Cog = factor of general cognitive ability; BMI = body mass index. Atrophy estimated with the ratio and residual method were flipped to match the difference score whereby larger values represent more brain atrophy. Note that different variables were extracted for the UKB sample in a way that maximised information across available time points, which means that the MRI scan and the ageing-related information is not necessarily assessed at the same time (see Methods). Some of the ageing-related measures in UKB used here were recorded prior to the MRI scan. We analyse all these measures as outcome variables to be predicted by LBA. Results describe their relationship, but we do not imply any directionality or causality. Percentages indicate variance explained (R^2) in the health trait, and is only printed if the association is statistically significant (p < 0.05/ 10 traits). Multinomial regressions treating APOEε4 as a categorical outcome variable yielded non-significant associations with LBA scores. Coefficients indicate the change in log-odds for having 1 or 2 APOEε4 relative to having 0 APOEε4 alleles. APOEε4 ~ LBA $_{residual}$: coefficient for having 1 allele relative to 0 alleles = -0.04 (SE = 0.13; p = 0.737); coefficient for having 2 alleles = -0.06 (SE = 0.13; p = 0.622). APOEε4 ~ LBA $_{ratio}$: coefficient for having 1 allele = -0.09 (SE = 0.13; p = 0.465); coefficient for having 2 alleles = -0.10 (SE = 0.13; p = 0.442). APOEε4 ~

LBA $_{difference}$: coefficient for having 1 allele = 0.18 (*SE* = 0.13; *p* = 0.162); coefficient for having 2 alleles = 0.17 (*SE* = 0.13; *p* = 0.203).

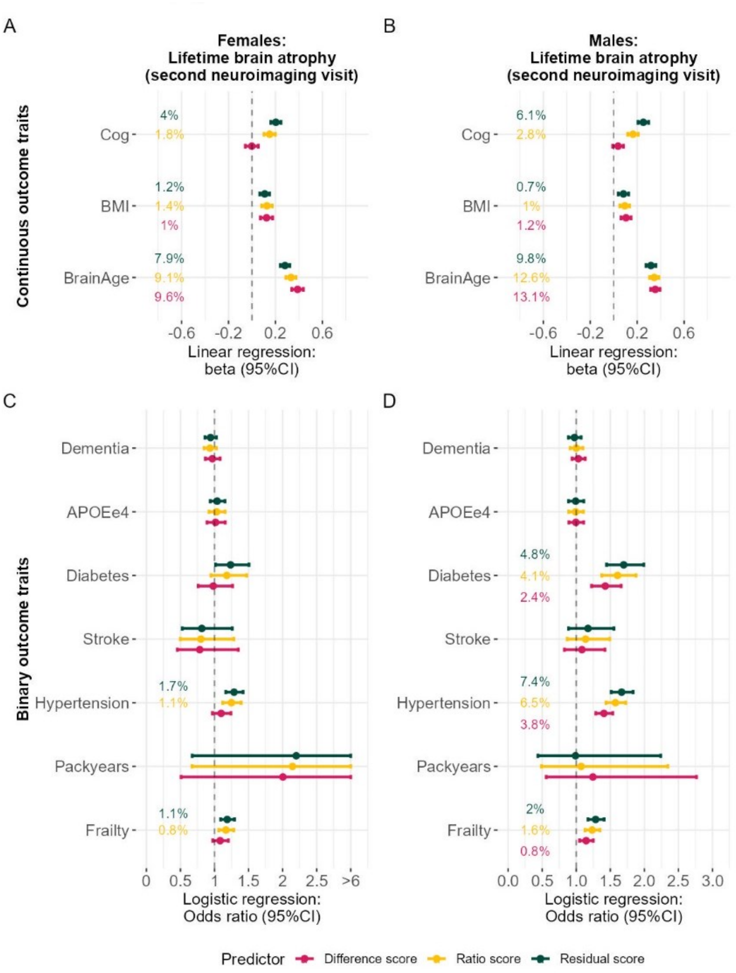

Fig.S8. Associations with health-related phenotypes in UKB for male (*n* = 2088) and female (*n* = 2265) sample separately. (A) Associations between LBA and continuous traits in females, (B) associations between LBA and continuous traits in males, (C) associations between LBA and binary traits in females, (D) associations between LBA and binary traits in males. Abbreviations: Cog = factor of general cognitive ability; BMI = body mass index. Residual scores were derived with references to male and female only reference groups. Atrophy estimated with the ratio and residual method were flipped to match the difference score whereby larger values represent more brain atrophy. Percentages indicate variance explained (R^2) in the health trait, and is only printed if the association is statistically significant ($p < 0.05/$ 10 traits). Note that different variables were extracted for the UKB sample in a way that maximised information across available time points, which means that the MRI scan and the ageing-related information is not necessarily assessed at the same time (see Supplementary Methods).

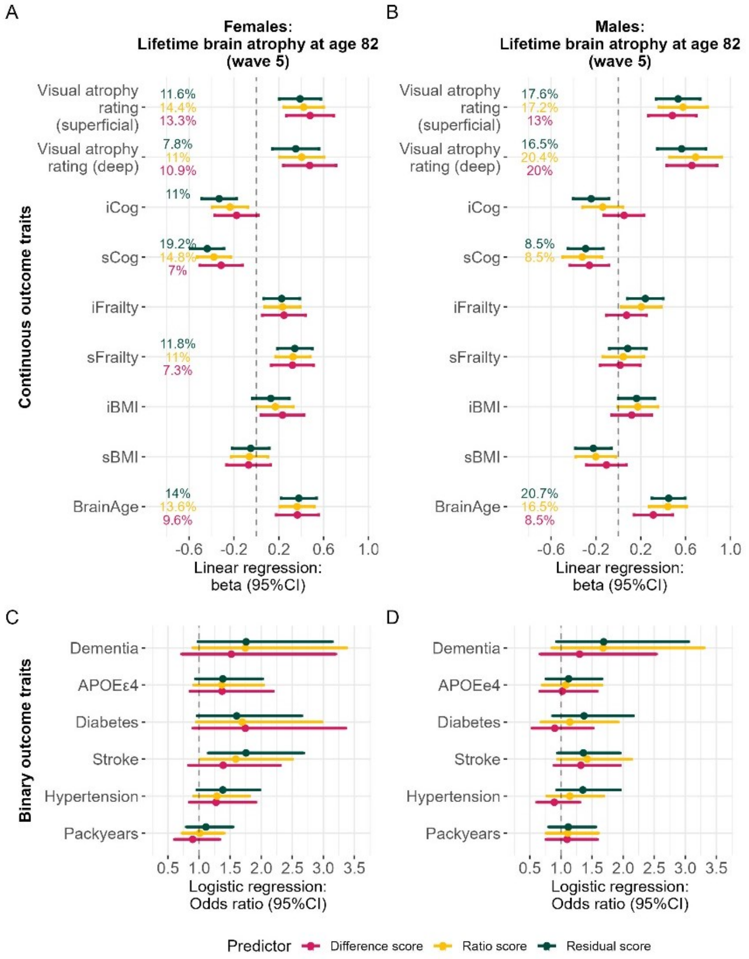

Fig.S9. Associations with health-related phenotypes in LBC1936 for male and female sample separately. (A) Associations between LBA and continuous traits in females, (B) associations between LBA and continuous traits in males, (C) associations between LBA and binary traits in females, (D) associations between LBA and binary traits in males. Abbreviations: iCog = intercept of growth curve model of cognitive tests across waves 1-5; sCog = slope of growth curve model of cognitive tests across waves 1-5; iFrailty & sFrailty = intercept and slope of growth curve model of frailty assessment across waves 1-5; iBMI & sBMI = intercept and slope of growth curve model of body mass index across waves 1-5. Residual scores were derived with references to male and female only reference groups. Atrophy estimated with the ratio and residual method were flipped to match the difference score whereby larger values represent more brain atrophy. Analyses presented here relied on the same participants as in *Fig.2*. Percentages indicate variance explained (R^2) in the health trait, and is only printed if the association is statistically significant (p < 0.05/ 15 traits).

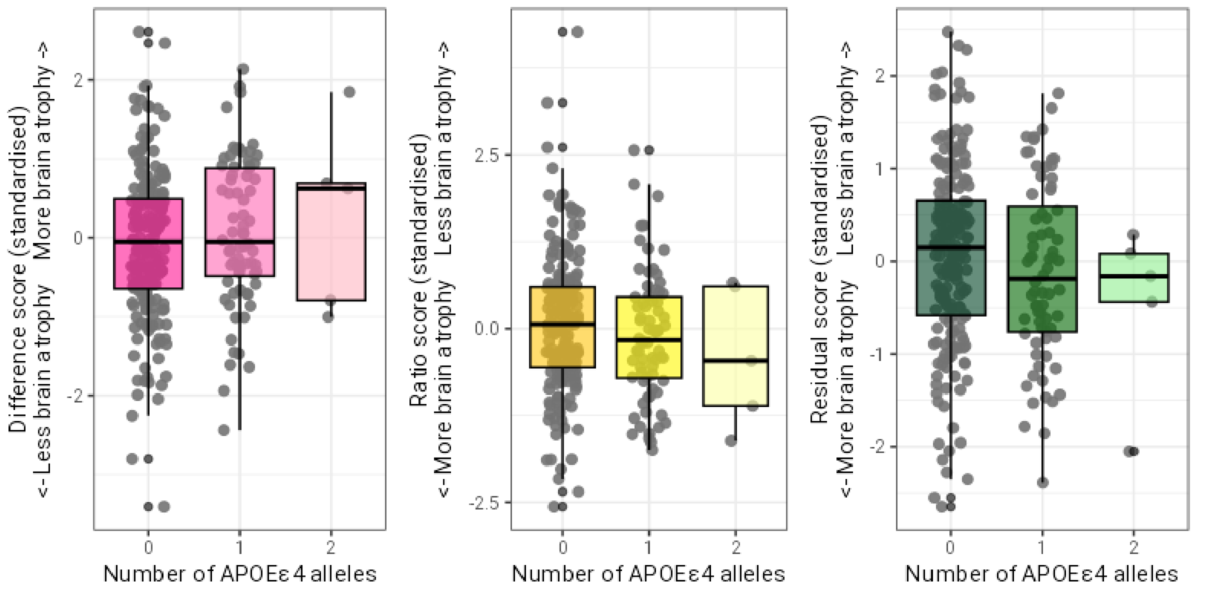

Fig.S10. LBC1936 ($N = 286$): Scatterplot showing LBA distributions by number of APOEε4 alleles. This data did not allow calculating multinomial regressions treating APOEε4 as a categorical outcome variable because it only had 5 participants in the group with two alleles.

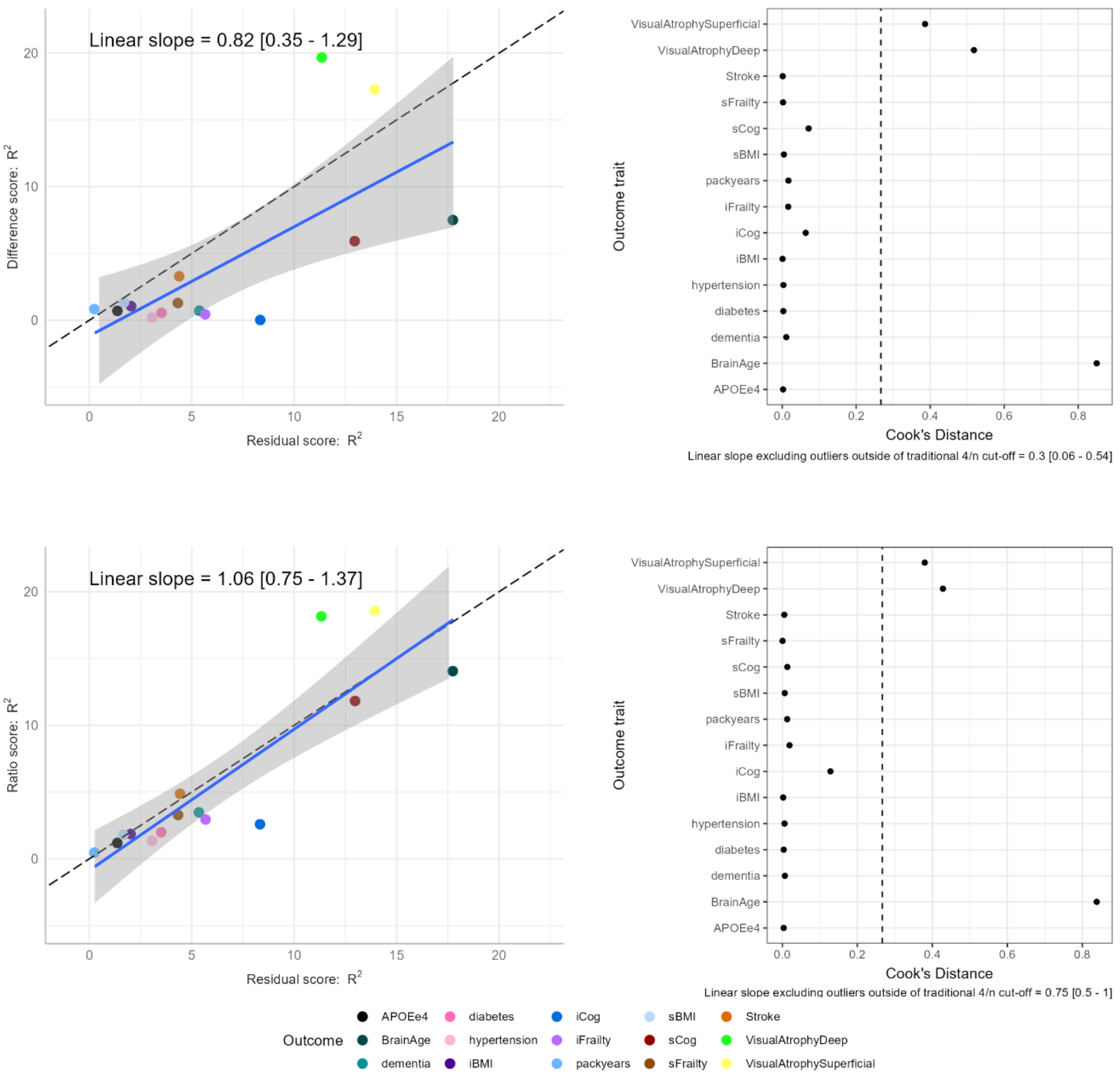

Fig.S11. LBC1936 (N = 286): Linear relationships between R^2 estimates displayed in *Fig.2* to contrast whether LBA $_{residual}$ predicted outcome traits consistently better than LBA $_{difference}$ or LBA $_{ratio}$ (left). Significance levels to determine whether the linear slope was significantly different from one were: $p = 0.454$ for LBA $_{difference}$ and $p = 0.723$ LBA $_{ratio}$ including outlier traits. Cook's distance (right) indicates outlier traits that negatively impacted the fit of the regression line. Significance levels to determine whether the linear slope was significantly different from one for regression excluding outlier traits were: $p = 0.0001$ for LBA $_{difference}$ and $p = 0.085$ LBA $_{ratio}$. Values in square brackets indicate 95% confidence intervals.

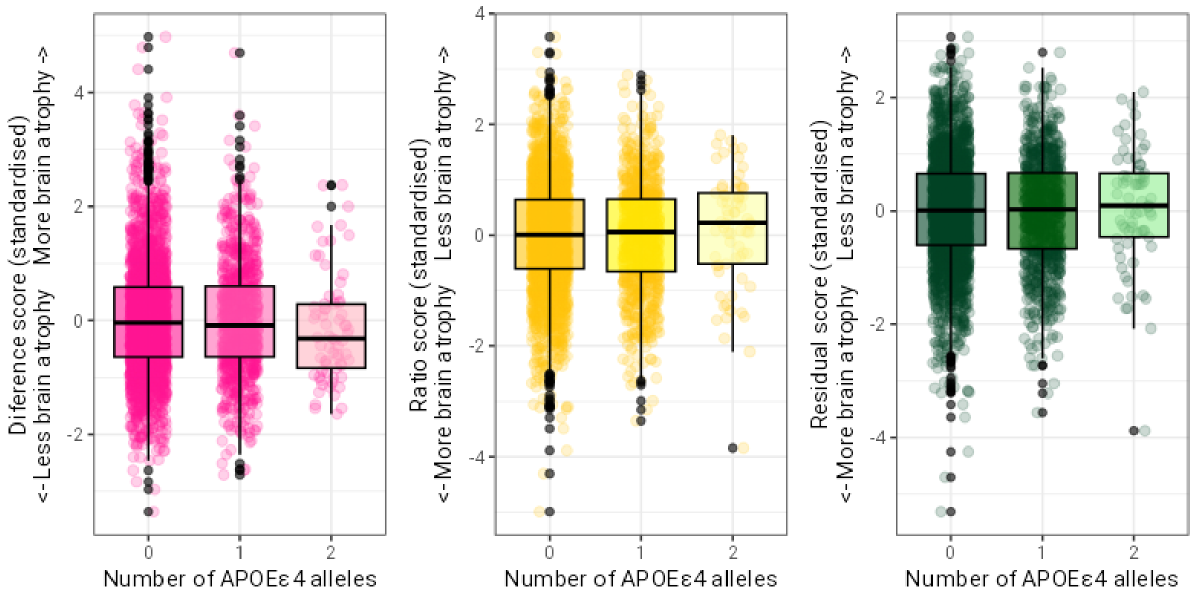

Fig.S12. UKB (*N* = 4006): LBA distributions displayed by number of APOEε4 alleles. Multinomial regressions treating APOEε4 as a categorical outcome variable yielded non-significant associations with LBA scores. Coefficients indicate the change in log-odds for having 1 or 2 APOEε4 relative to having 0 APOEε4 alleles. APOEε4 ~ LBA $_{residual}$: coefficient for having 1 allele relative to 0 alleles = -0.04 (*SE* = 0.13; *p* = 0.737); coefficient for having 2 alleles = -0.06 (*SE* = 0.13; *p* = 0.622). APOEε4 ~ LBA $_{ratio}$: coefficient for having 1 allele = -0.09 (*SE* = 0.13; *p* = 0.465); coefficient for having 2 alleles = -0.10 (*SE* = 0.13; *p* = 0.442). APOEε4 ~ LBA $_{difference}$: coefficient for having 1 allele = 0.18 (*SE* = 0.13; *p* = 0.162); coefficient for having 2 alleles = 0.17 (*SE* = 0.13; *p* = 0.203).

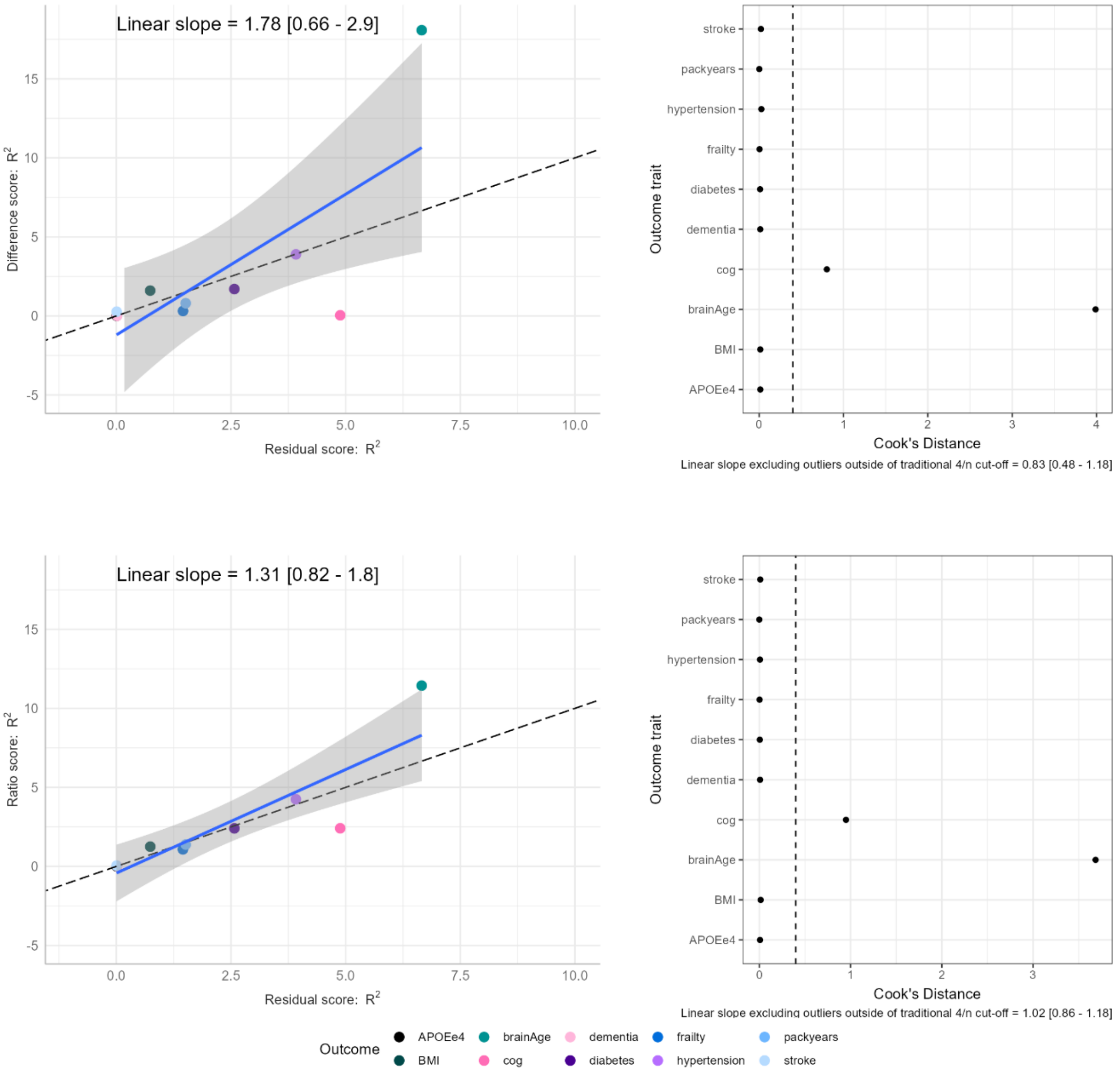

Fig.S13. UKB (N = 4674): Linear relationships between R^2 estimates displayed in *Fig.2* to contrast whether LBA$_{\text{residual}}$ predicted outcome traits consistently better than LBA$_{\text{difference}}$ or LBA$_{\text{ratio}}$ (left). Significance levels to determine whether the linear slope was significantly different from one were: $p = 0.211$ for LBA$_{\text{difference}}$ and $p = 0.253$ LBA$_{\text{ratio}}$ including outlier traits. Cook's distance (right) indicates outlier traits that negatively impacted the fit of the regression line. Significance levels to determine whether the linear slope was significantly different from one for regression excluding outlier traits were: $p = 0.832$ for LBA$_{\text{difference}}$ and $p = 0.384$ LBA$_{\text{ratio}}$. Values in square brackets indicate 95% confidence intervals.

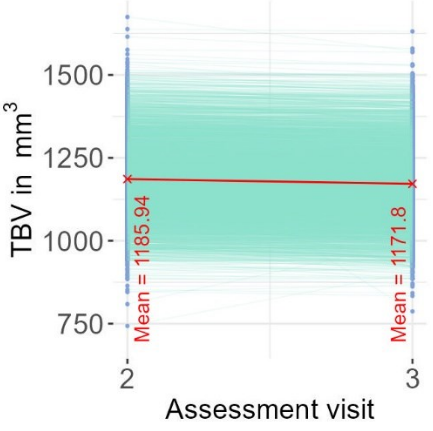

Fig.S14. TBV estimates (*mm*^{3}) in UKB participants with two MRI scans; *N* = 4682 at initial and repeated neuroimaging visit, mean lag = 4 years, age range at baseline = 46-81 years.

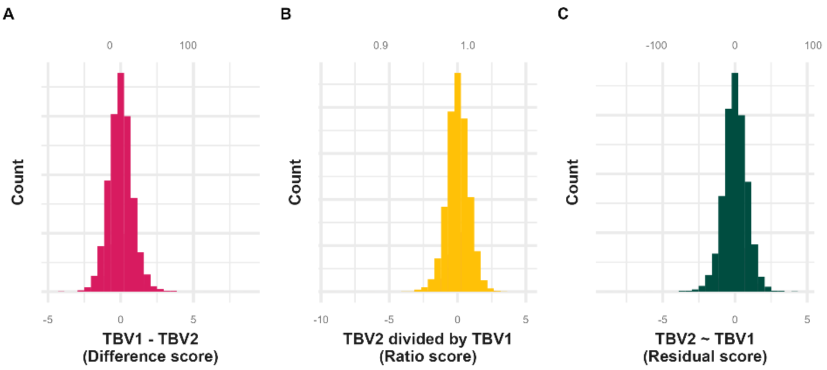

Fig.S15. Distribution of atrophy scores derived from repeated TBV estimates in UKB (*N* = 4674). LBA $_{\text{difference score}}$: minimum = -4.6, maximum = 8.55; LBA $_{\text{ratio score}}$: minimum = -9.03, maximum = 5.02; LBA $_{\text{residual score}}$: minimum = -8.72, maximum = 4.56.

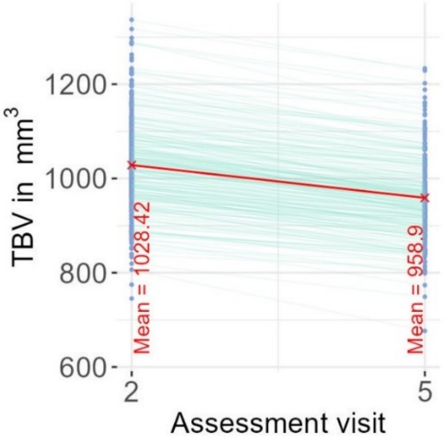

Fig.S16. TBV estimates (*mm*^{3}) in LBC1936 (N = 286) at first scan (wave 2) and fourth scan (wave 5), mean lag = 9 years, age range at baseline = 71-74 years.

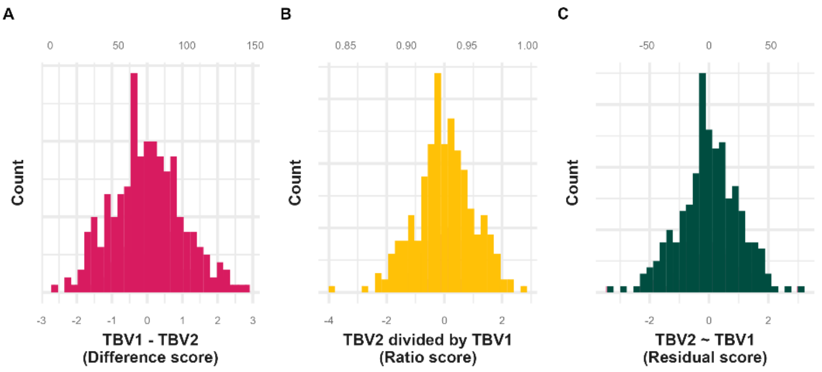

Fig.S17. Distribution of atrophy scores derived from repeated TBV estimates in LBC1936 (N = 4674). (A) Difference score, (B) ratio score, (C) residual score.Top x-axis: raw values; bottom x-axis: standardised value. LBA $_{\text{difference score}}$: minimum = -2.63, maximum = 2.81; LBA $_{\text{ratio score}}$: minimum = -3.88, maximum = 2.77; LBA $_{\text{residual score}}$: minimum = -3.43, maximum = 3.00.

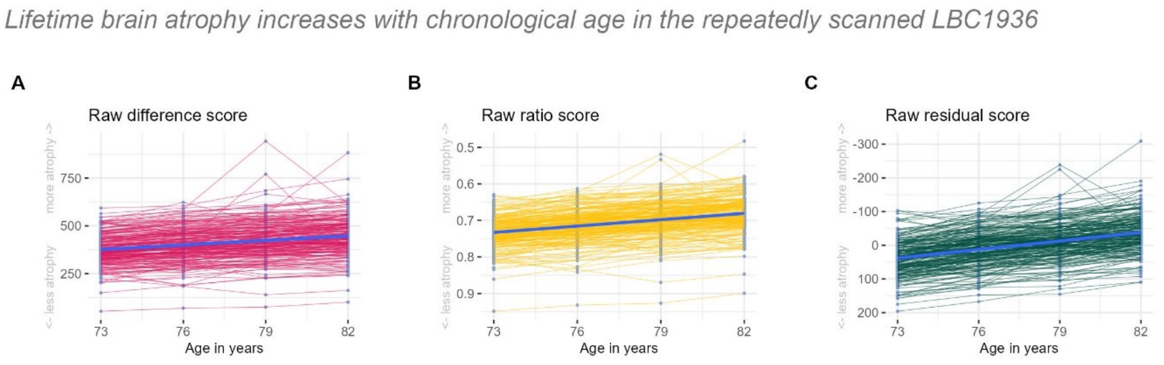

Fig.S18. Repeated MRI measures age-correlation. (A) Difference score, (B) ratio score, (C) residual score. LBA inferred at four assessments in the LBC1936 (N = 286). Y-axis shows raw value for each of the scores. LBA was inferred at each time point from TBV and ICV estimates processed with the cross-sectional FS stream, which was meant to illustrate the tendency of LBA to increase with age, even when ICV is not specifically held constant across time points (which is what the FS longitudinal processing stream would have achieved). This figure displays only participants with estimates available across all waves. In this figure the residual score was calculated based on measurements at 4 time points so that atrophy estimates across all time points were derived relative to the same average value (this was required to illustrate time-dependent increases). In contrast, all other analyses across the manuscript derived the residual score based on one individual visit with only one entry for each participant. This figure displays raw LBA scores where ratio and residual scores were *not* flipped (i.e., multiplied with -1), but their *y*-axes are reversed. The scores were, however, flipped in all other figures and analyses to represent more LBA with larger values, and less LBA with smaller values.

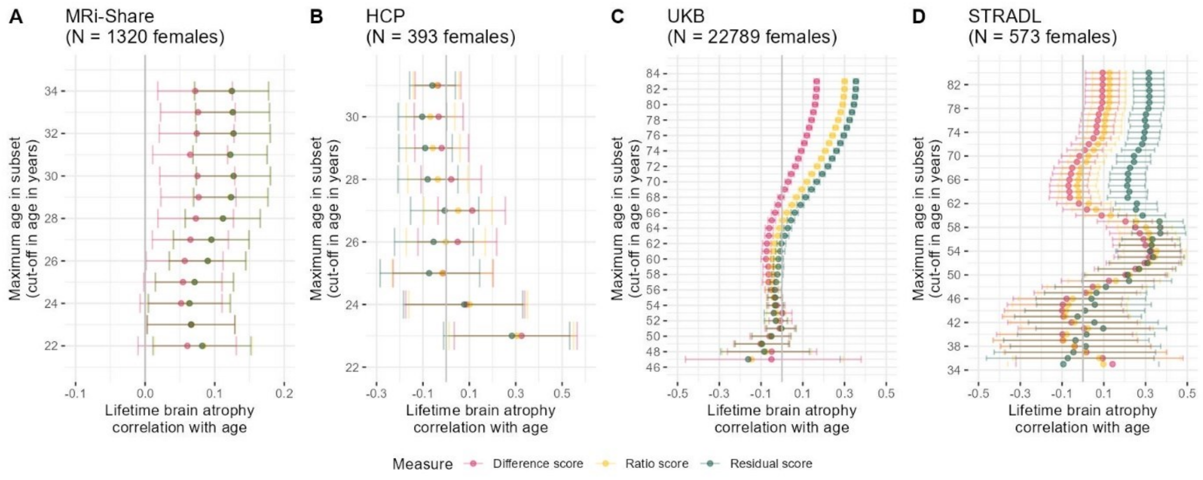

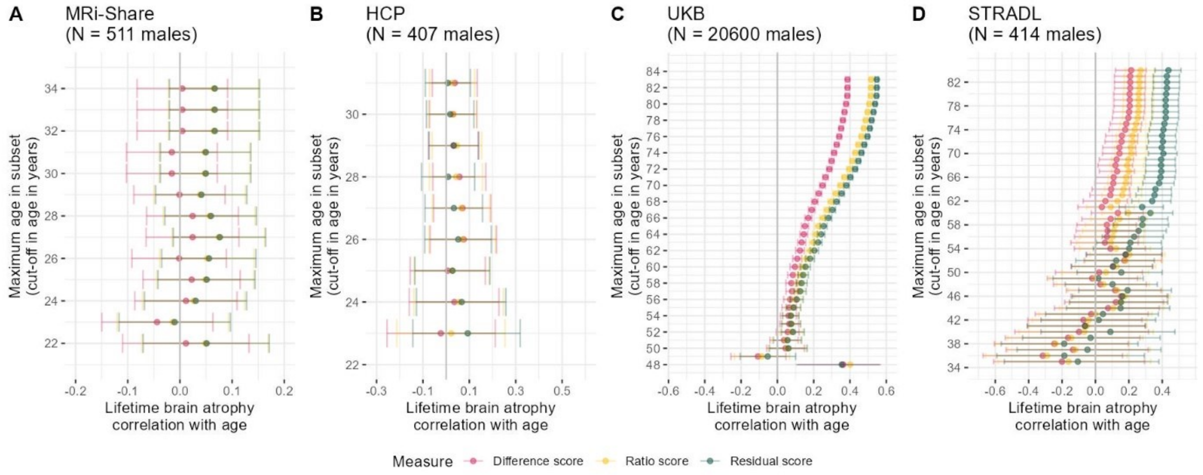

Fig.S19. LBA is moderated by sample age across four earlier- and later-life cohorts. (A) MRi-Share, (B) HCP, (C) UKB, (D) STRADL – Generation Scotland. Re-analysis of data presented in Fig.3 split into males and females separately. Abbreviations: HCP = Human Connectome project, UKB = UK Biobank, STRADL is the Generation Scotland neuroimaging subsample.

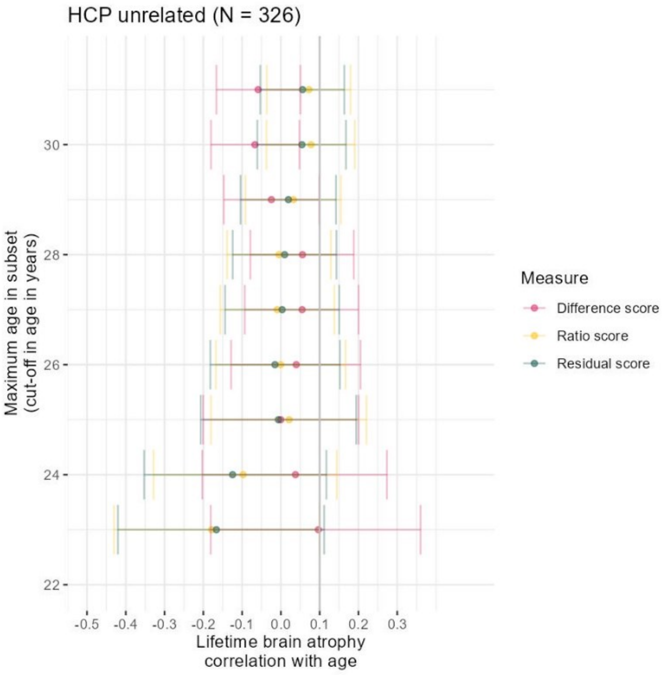

Fig.S20. Age correlations in unrelated Human Connectome Project (HC) sample (same as *Fig.2*, only in reduced unrelated sample). X-axis displays the correlation between LBA and age. Y-axis shows the maximum sample age that was used to extract the subsample. For example, when it says 30 on the y-axis, it means that this data point was calculated in a subsample of HCP where participants were aged 30 and under.

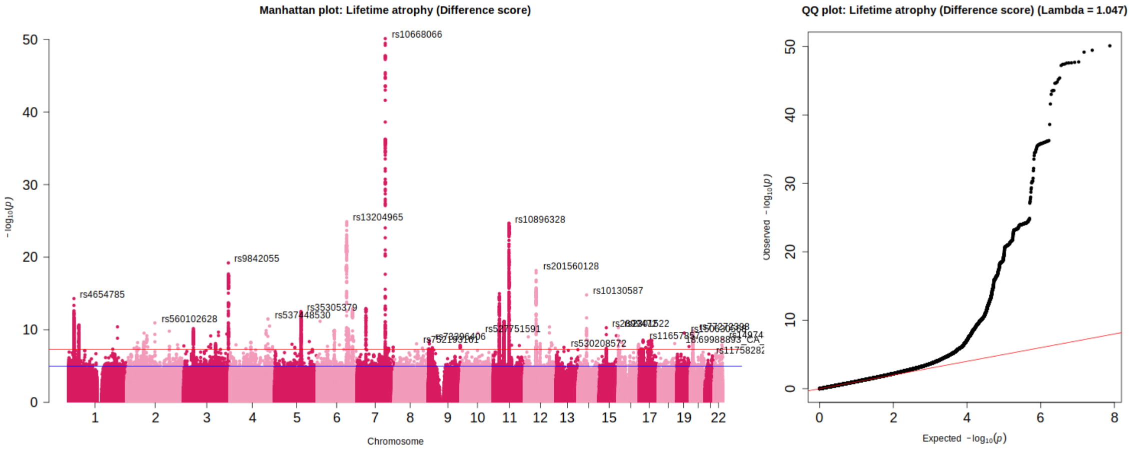

Fig.S21. Manhattan (left) and quantile-quantile plot (right) for lifetime brain atrophy inferred with the difference method (*N* = 43,110). Single nucleotide polymorphisms labelled in the Manhattan plot were the top GWAS hits. Lambda indicates the genomic inflation factor.

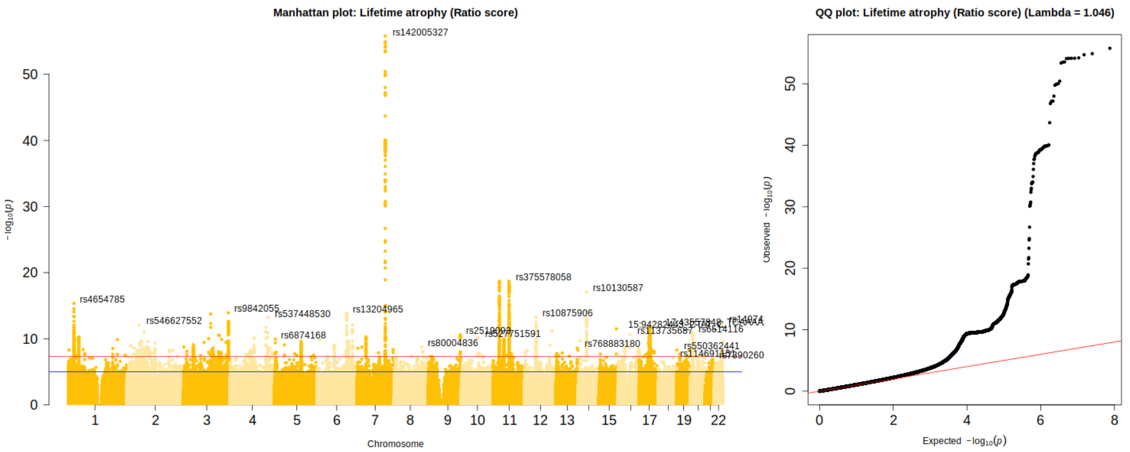

Fig.S22. Manhattan (left) and quantile-quantile plot (right) for lifetime brain atrophy inferred with the ratio method (*N* = 43,110). Single nucleotide polymorphisms labelled in the Manhattan plot were the top GWAS hits. Lambda indicates the genomic inflation factor.

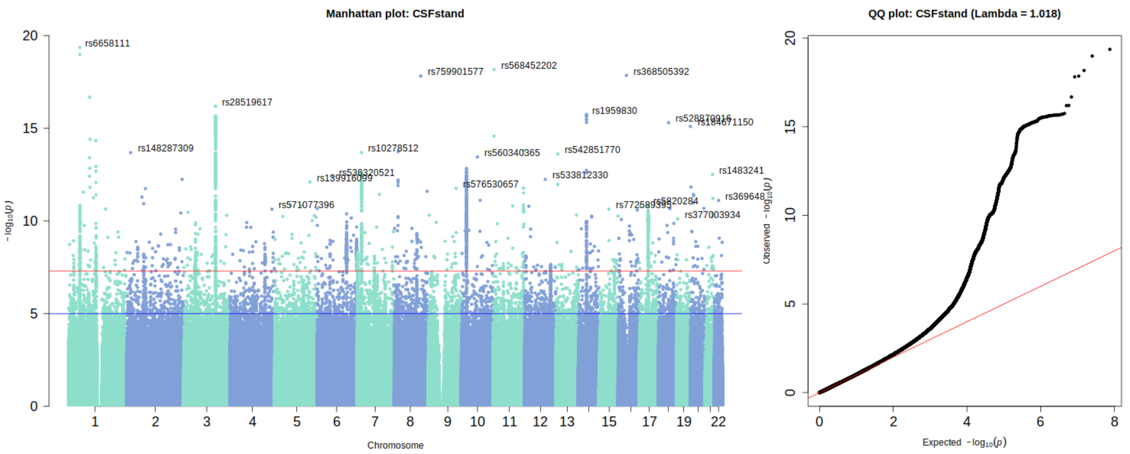

Fig.S23. Manhattan (left) and quantile-quantile plot (right) for cerebrospinal fluid (CSF) volume (*N* = 43,110). Single nucleotide polymorphisms labelled in the Manhattan plot were the top GWAS hits. Lambda indicates the genomic inflation factor.

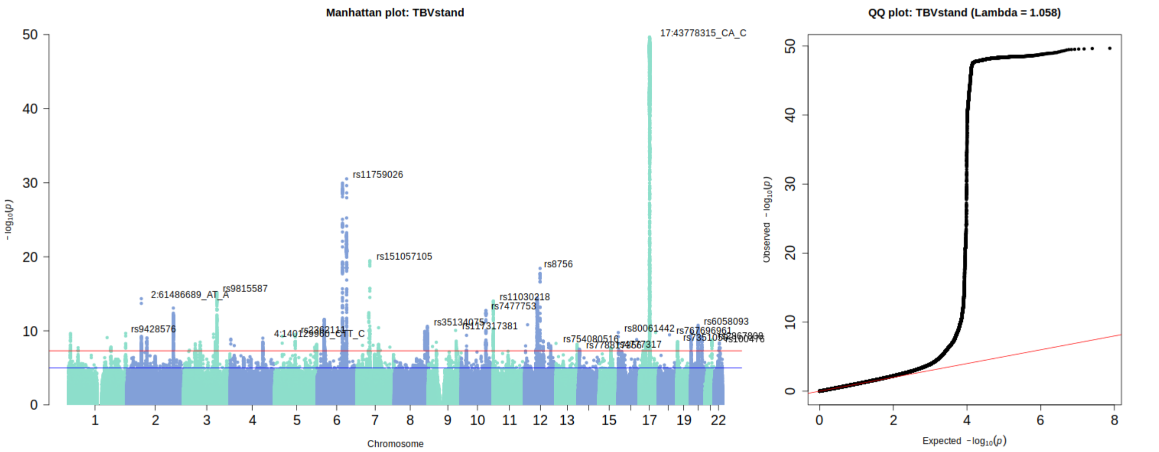

Fig.S24. Manhattan (left) and quantile-quantile plot (right) for total brain volume (TBV; $N = 43{,}110$). Single nucleotide polymorphisms labelled in the Manhattan plot were the top GWAS hits. Lambda indicates the genomic inflation factor.

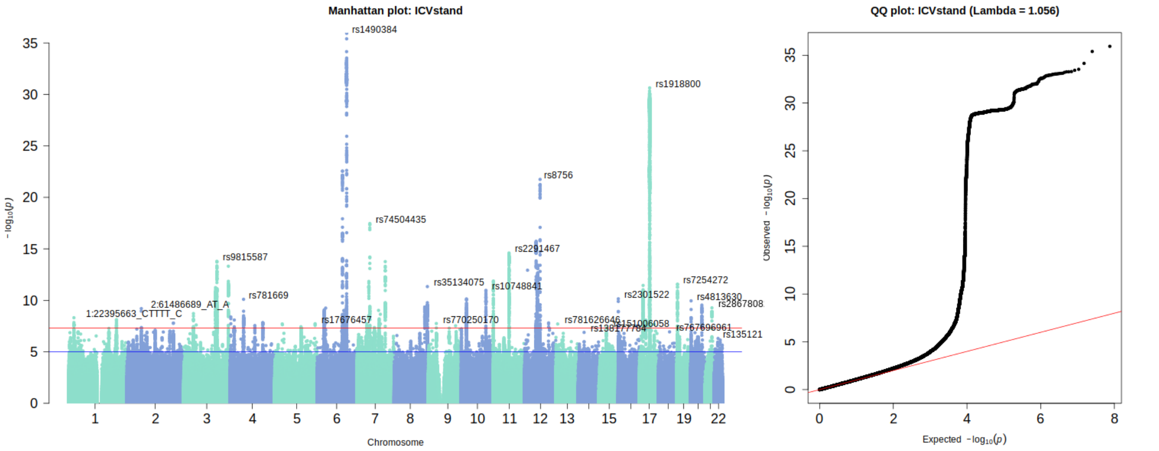

Fig.S25. Manhattan (left) and quantile-quantile plot (right) for intracranial volume (ICV; $N = 43{,}110$). Single nucleotide polymorphisms labelled in the Manhattan plot were the top GWAS hits. Lambda indicates the genomic inflation factor.

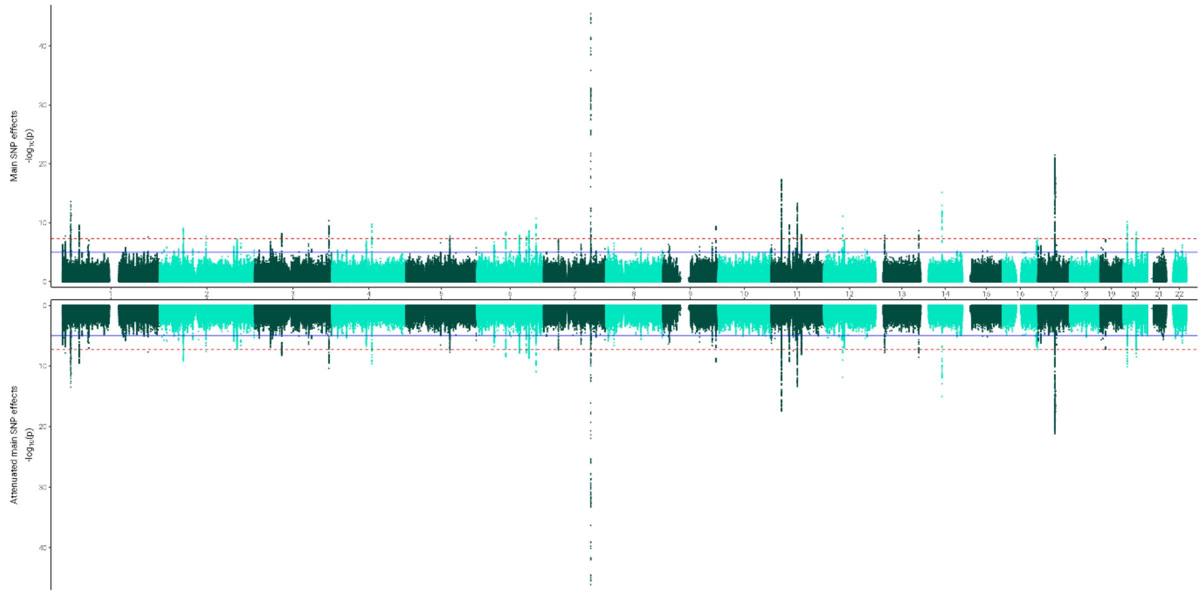

Fig.S26. Miami plots for **residual score** contrasting main SNP effect *p*-values from original GWAS with main SNP effect *p*-values from GWAS analyses (*N* = 43,110) including an age interaction term (SNP filters: MAF > 0.01, INFO > 0.9, diallelic)

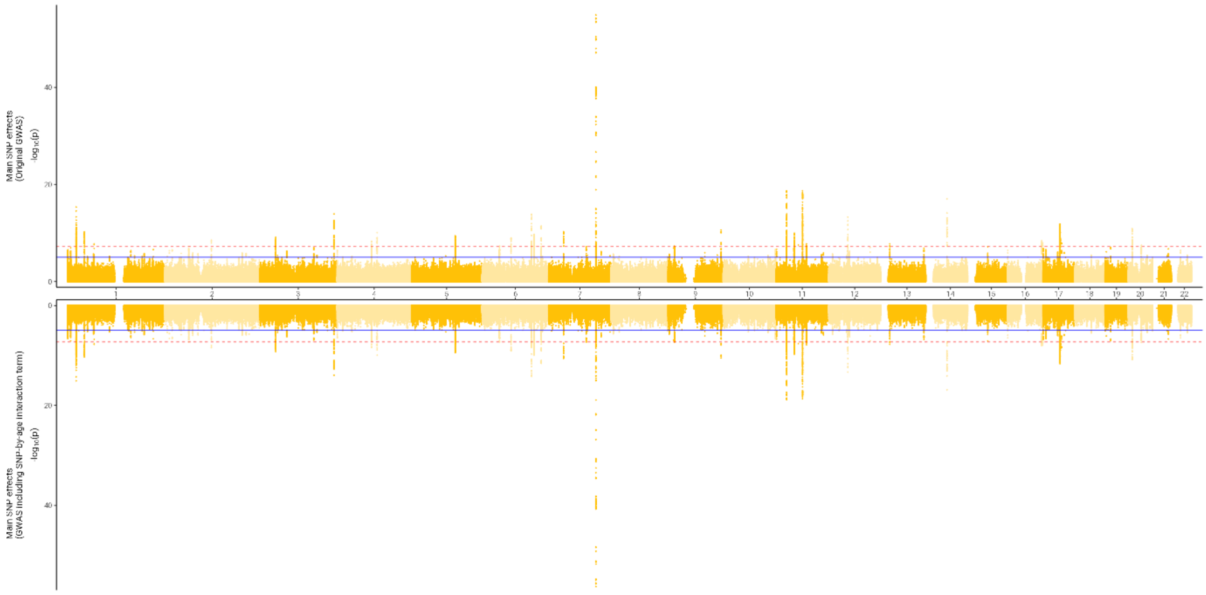

Fig.S27. Miami plots for **ratio score** contrasting main SNP effect *p*-values from original GWAS with main SNP effect *p*-values from GWAS analyses (*N* = 43,110) including an age interaction term (SNP filters: MAF > 0.01, INFO > 0.9, diallelic)

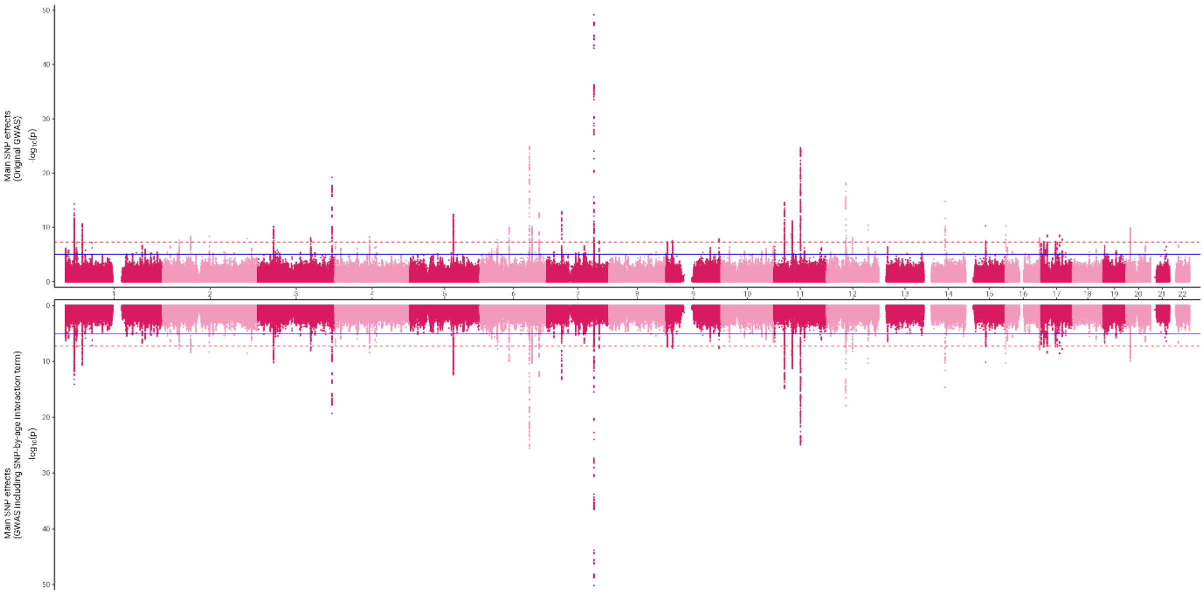

Fig.S28. Miami plots for **difference score** contrasting main SNP effect *p*-values from original GWAS with main SNP effect *p*-values from GWAS analyses (*N* = 43,110) including an age interaction term (SNP filters: MAF > 0.01, INFO > 0.9, diallelic)

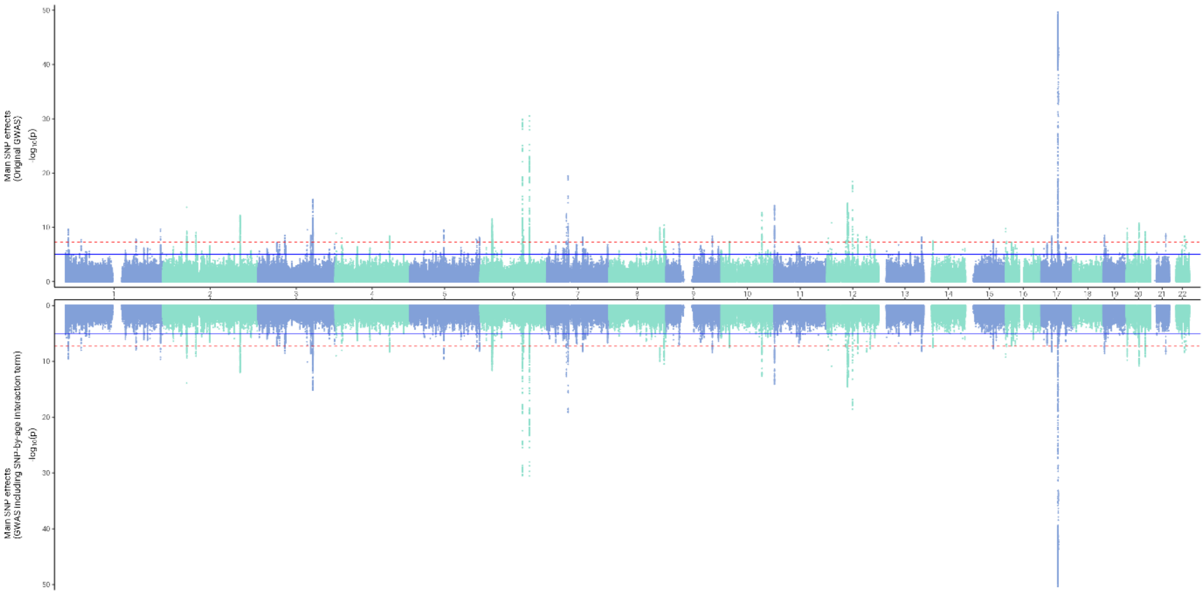

Fig.S29. Miami plots for **TBV** contrasting main SNP effect *p*-values from original GWAS with main SNP effect *p*-values from GWAS analyses (*N* = 43,110) including an age interaction term (SNP filters: MAF > 0.01, INFO > 0.9, diallelic)

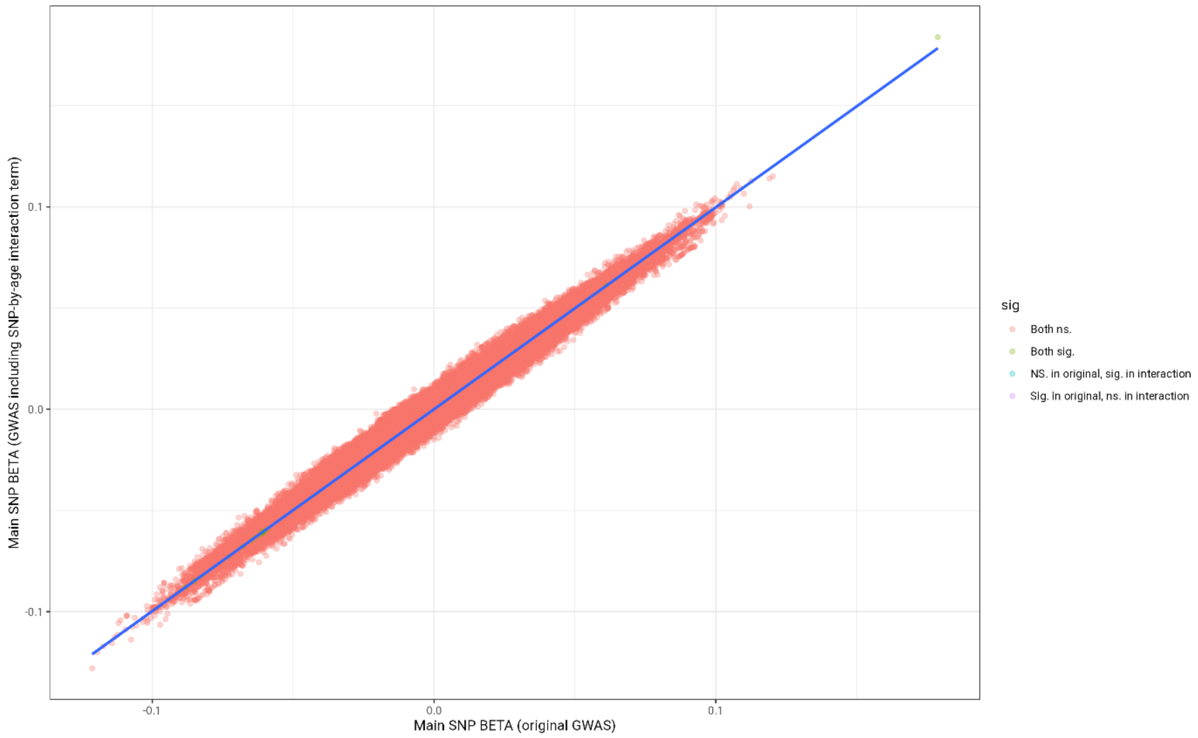

Fig.S30. **Residual score**: Contrasting main SNP beta effect sizes from original GWAS with main SNP beta effect sizes from GWAS analyses (N = 43,110) including an age interaction term (SNP filters: MAF > 0.01, INFO > 0.9, diallelic)

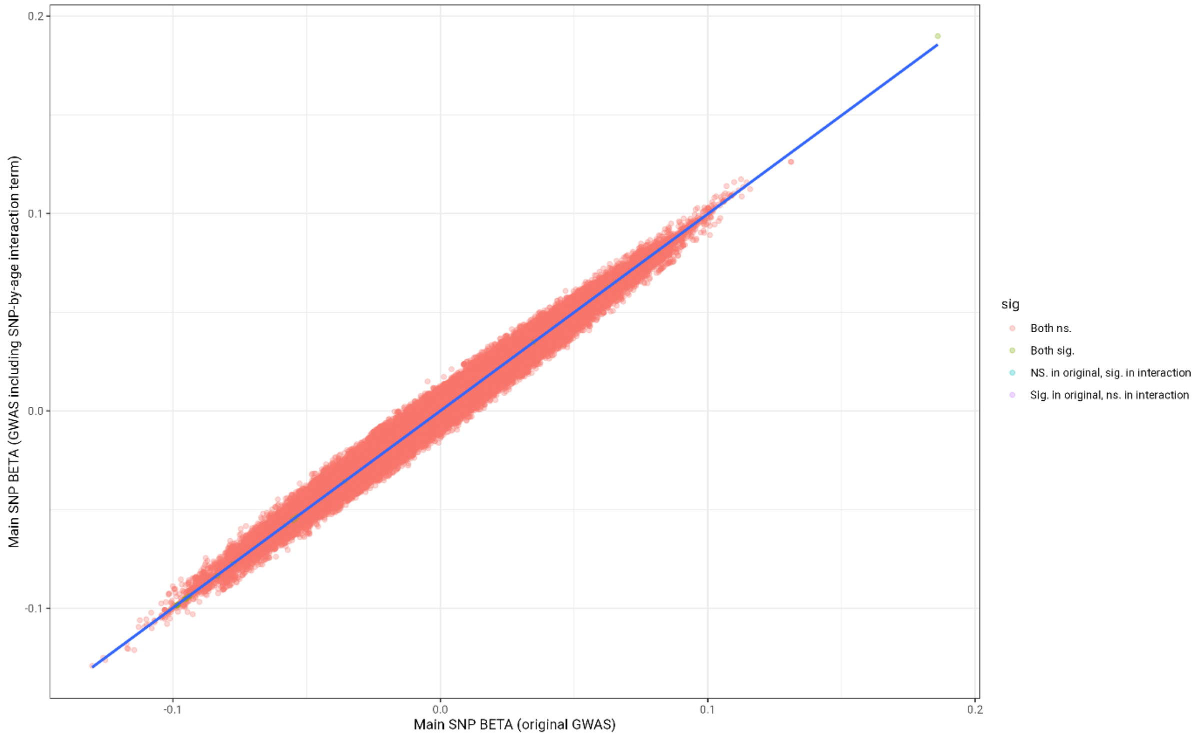

Fig.S31. **Ratio score**: Contrasting main SNP beta effect sizes from original GWAS with main SNP beta effect sizes from GWAS analyses (N = 43,110) including an age interaction term (SNP filters: MAF > 0.01, INFO > 0.9, diallelic)

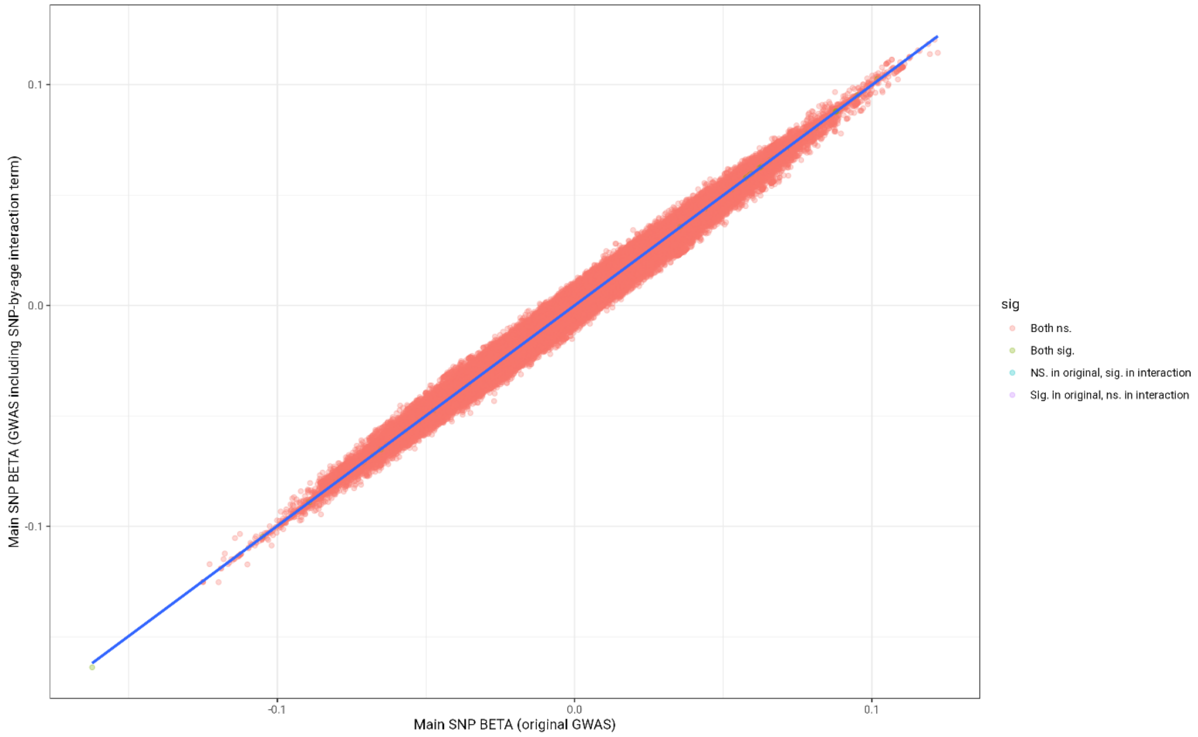

Fig.S32. **Difference score**: Contrasting main SNP beta effect sizes from original GWAS with main SNP beta effect sizes from GWAS analyses (N = 43,110) including an age interaction term (SNP filters: MAF > 0.01, INFO > 0.9, diallelic)

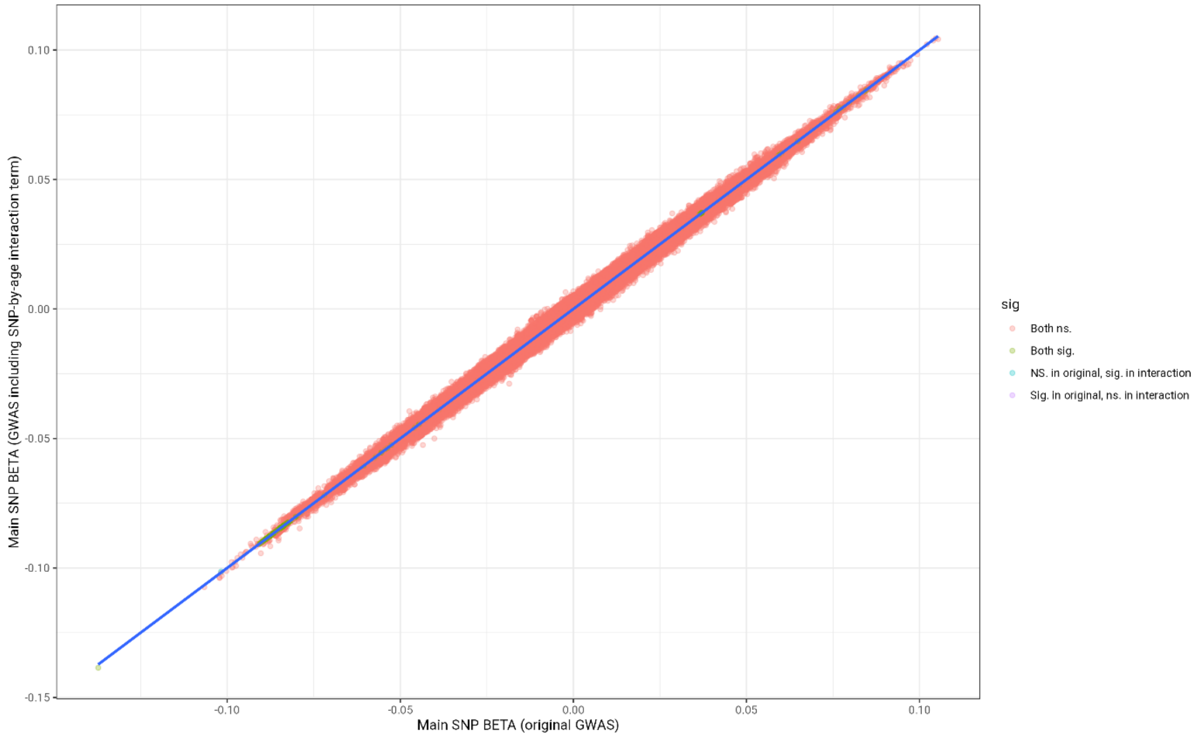

Fig.S33. **TBV**: Contrasting main SNP beta effect sizes from original GWAS with main SNP beta effect sizes from GWAS analyses (*N* = 43,110) including an age interaction term (SNP filters: MAF > 0.01, INFO > 0.9, diallelic)

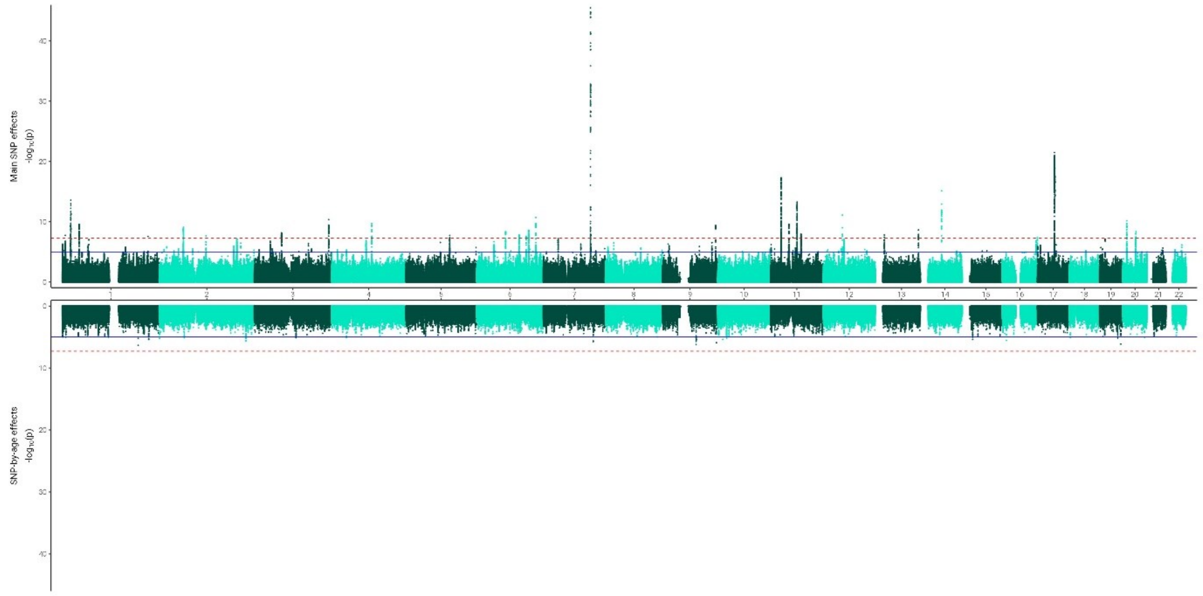

Fig.S34. Miami plots for **residual score** contrasting main SNP effect *p*-values from original GWAS with SNP-by-age interaction *p*-values from GWAS analyses (*N* = 43,110) including an age interaction term (SNP filters: MAF > 0.01, INFO > 0.9, diallelic)

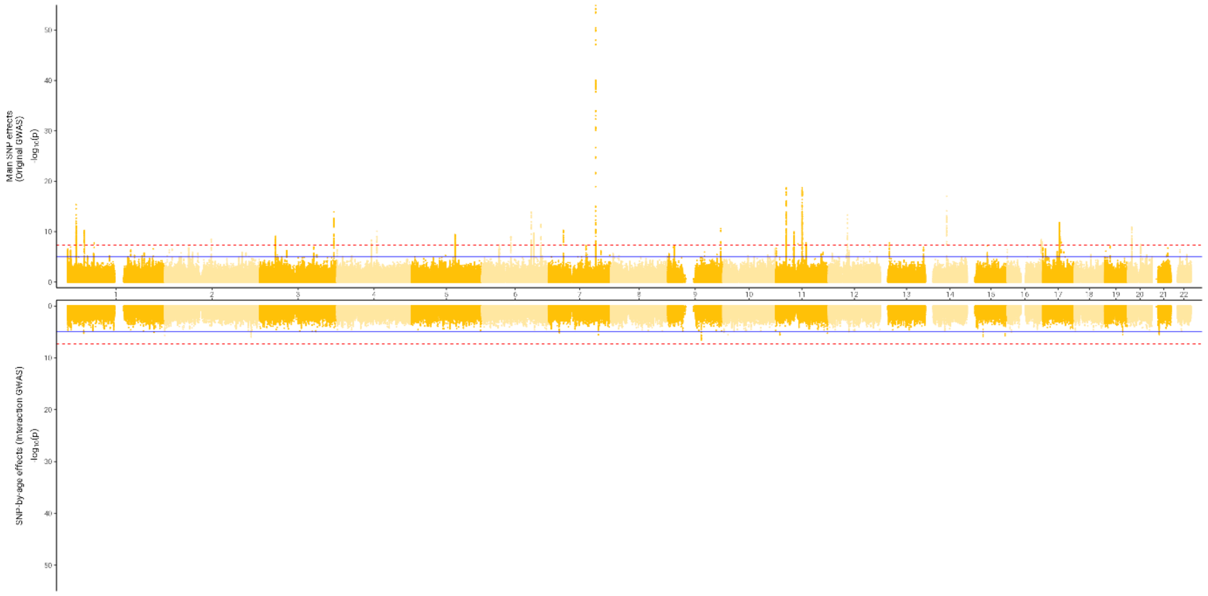

Fig.S35. Miami plots for **ratio score** contrasting main SNP effect *p*-values from original GWAS with SNP-by-age interaction *p*-values from GWAS analyses (*N* = 43,110) including an age interaction term (SNP filters: MAF > 0.01, INFO > 0.9, diallelic)

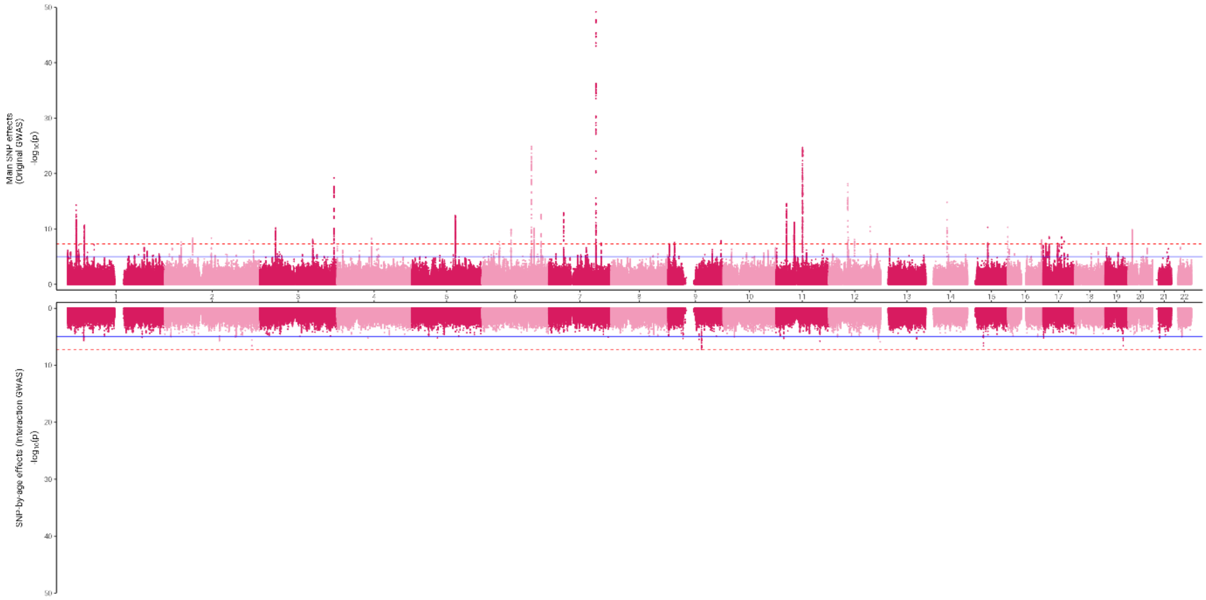

Fig.S36. Miami plots for **difference score** contrasting main SNP effect *p*-values from original GWAS with SNP-by-age interaction *p*-values from GWAS analyses (*N* = 43,110) including an age interaction term (SNP filters: MAF > 0.01, INFO > 0.9, diallelic)

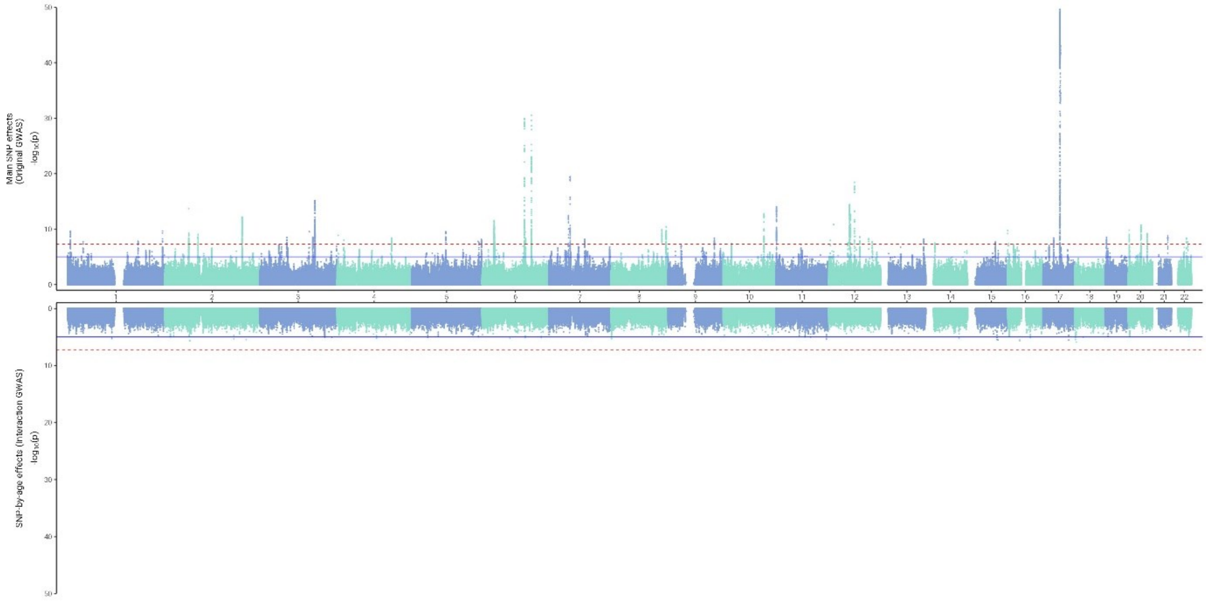

Fig.S37. Miami plots for **TBV** contrasting main SNP effect *p*-values from original GWAS with SNP-by-age interaction *p*-values from GWAS analyses (*N* = 43,110) including an age interaction term (SNP filters: MAF > 0.01, INFO > 0.9, diallelic)

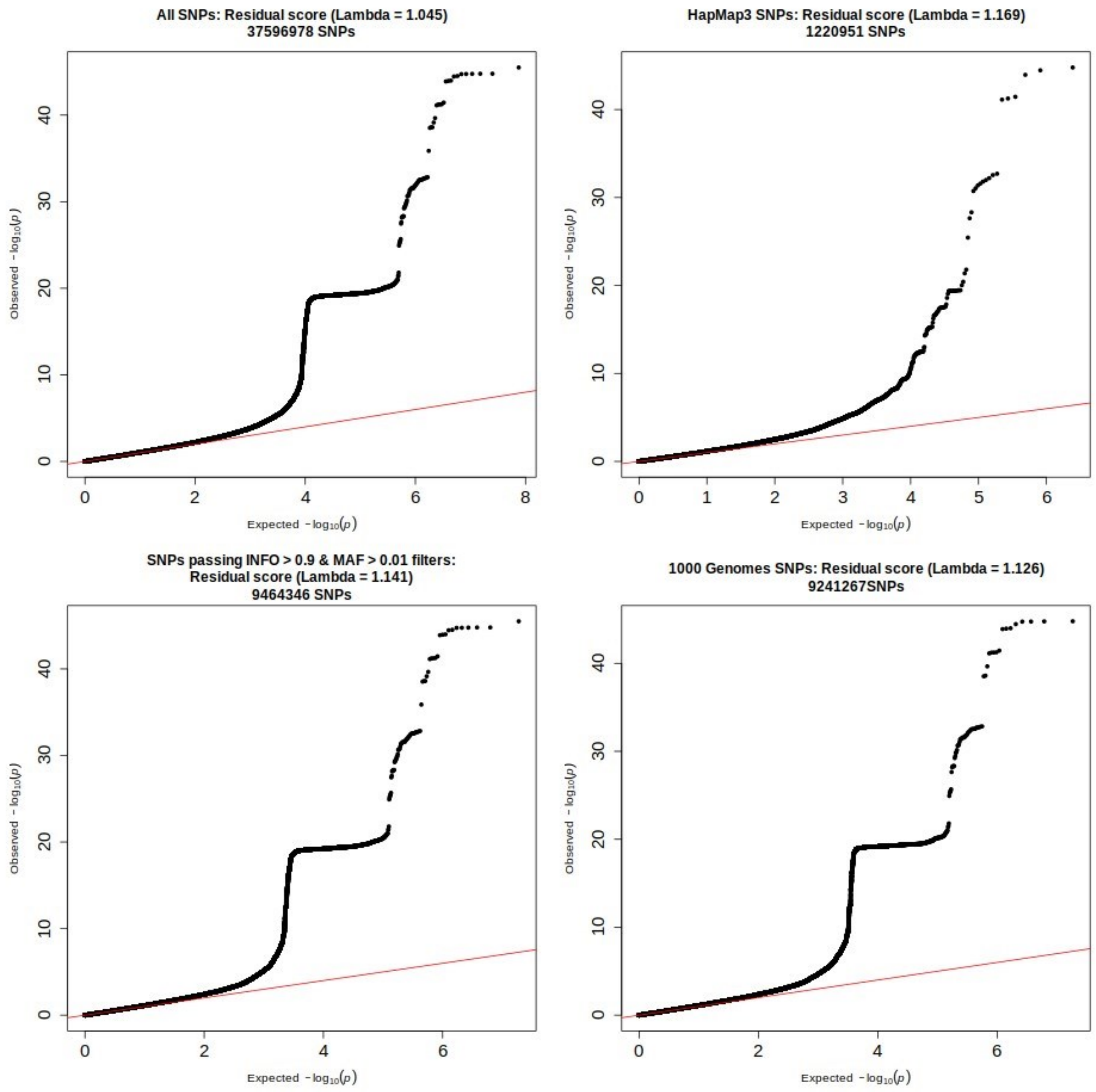

Fig.S38. QQ plots for residual score including **top left:** all SNPs, **top right:** only HapMap3 SNPs, **bottom left:** SNPs that pass an INFO filter > 0.9, and **bottom right:** MAF filter > 0.1, and 1000 Genomes SNPs. The step in the QQ plot, often present in GWAS (N = 43,110) QQ plots of neuroimaging phenotypes, disappears when considering HapMap SNPs only

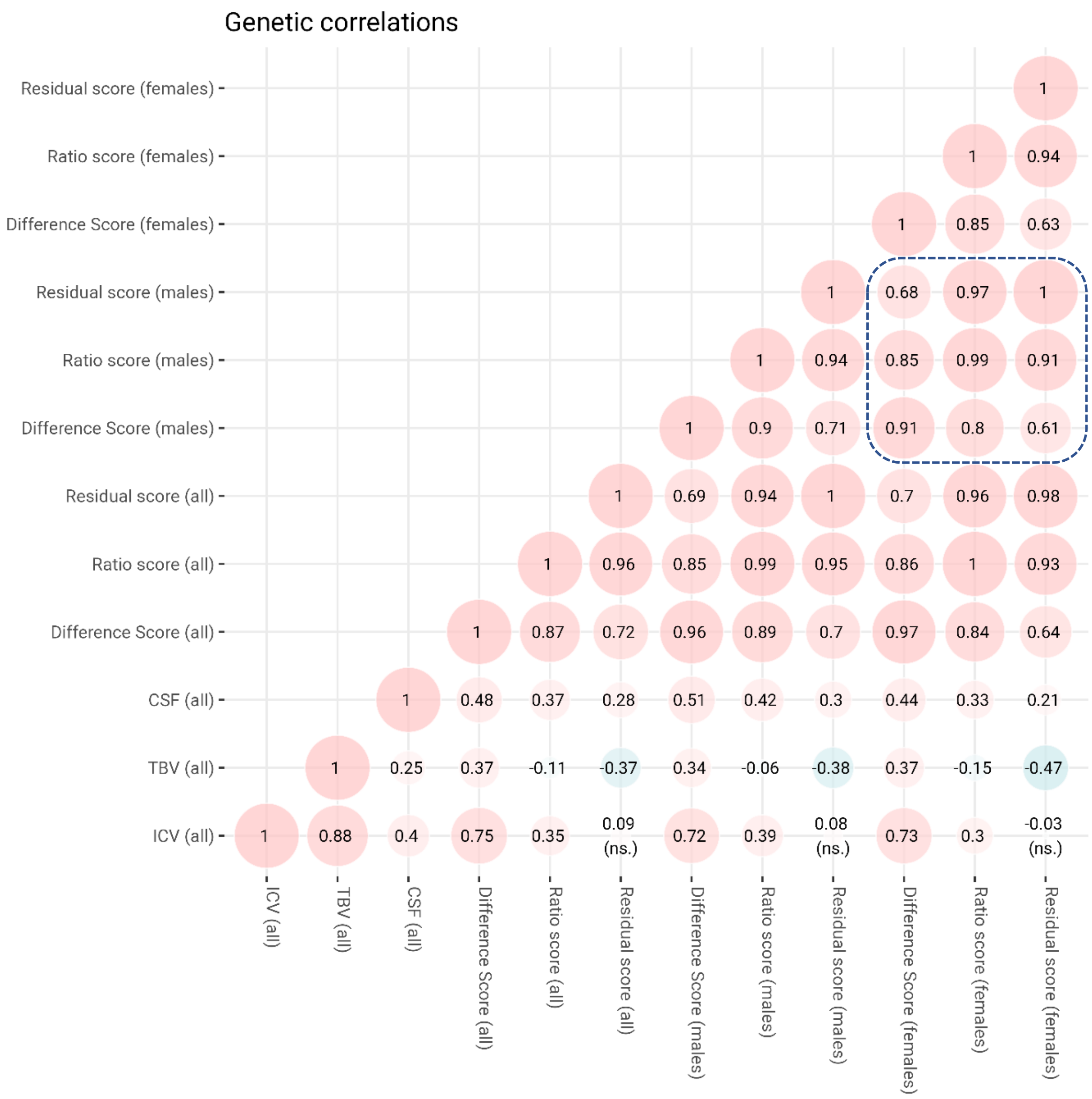

Fig.S39. Genetic correlations calculated from GWAS summary statistics in the whole UKB sample (all; $N = 43{,}110$), males only ($N = 20{,}453$), and females only ($N = 22{,}657$). Dotted box highlights male-female genetic correlations. LDSC heritability (SE) were $LBA_{difference} = 0.37$ (0.03), $LBA_{ratio} = 0.30$ (0.03), $LBA_{residual} = 0.27$ (0.03) for males, and $LBA_{difference} = 0.27$ (0.03), $LBA_{ratio} = 0.24$ (0.03), $LBA_{residual} = 0.25$ (0.03) for females.

Comparing residual (two-step approach) and adjustment method (one-step approach)

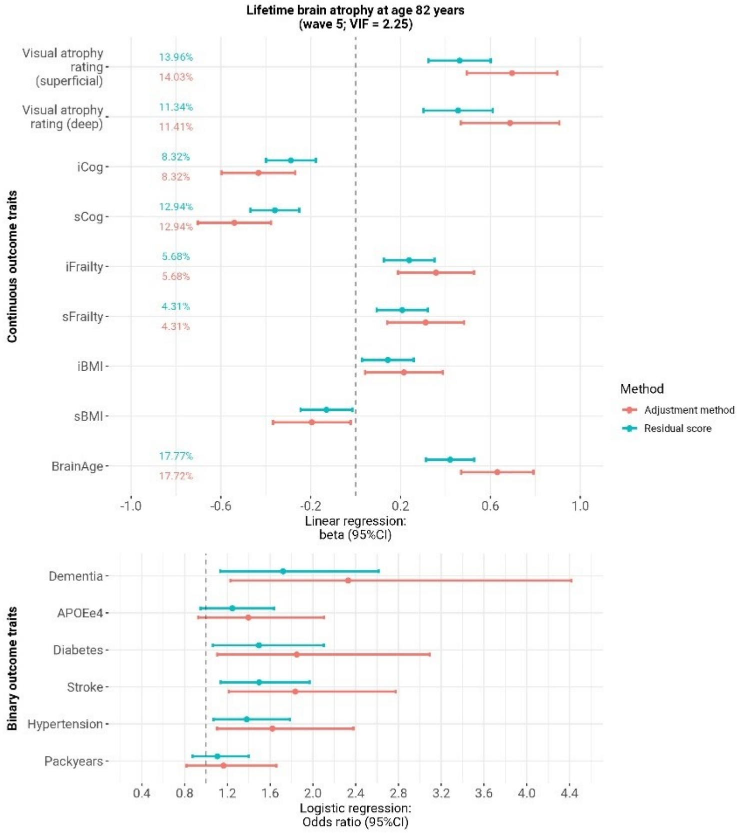

Fig.S40. Associations with health-related phenotypes in LBC1936 for LBA $_{residual}$ compared with the same associations for one-step adjustment method ($N = 286$). LBA $_{residual}$ was modelled in a previous regression; *lm(TBV~ICV)*, before testing its association with health-related phenotypes; *lm(pheno ~ LBA $_{residual}$)*. The adjustment method is set out to model the same thing but in one step; *lm(pheno ~ TBV + ICV)*. VIF was calculated with the car::vif() function in R, and is unchanged across all these models because it is calculated based on the independent variables which are the same across those models. The unique contribution to R^2 by TBV in the adjustment method was calculated by subtracting the R^2 of a model excluding TBV from the full models' R^2.

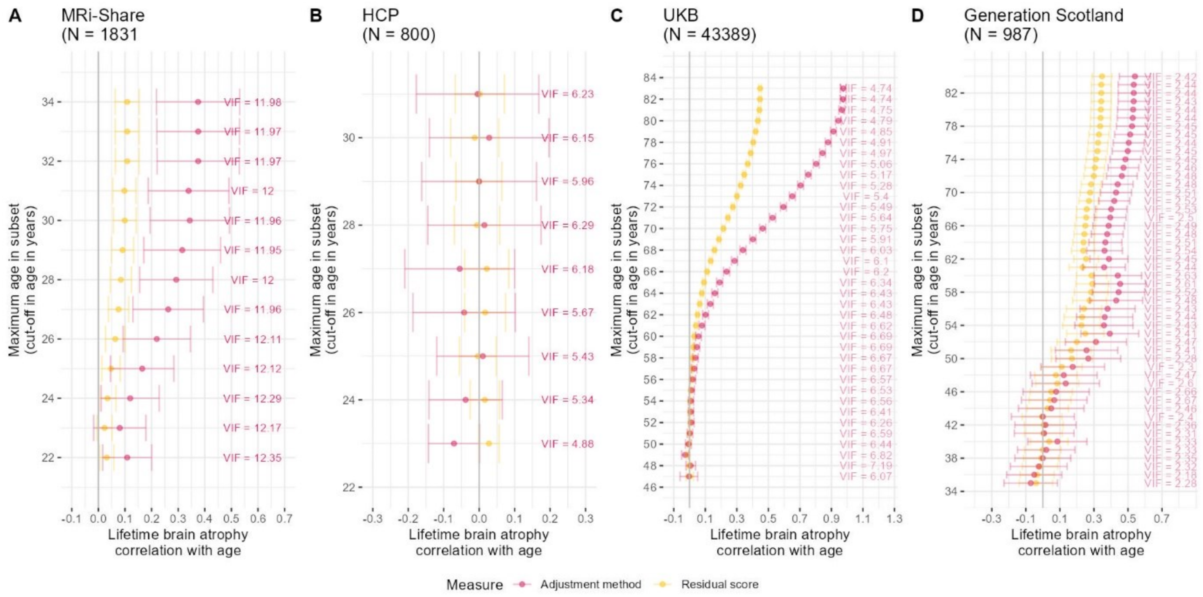

Fig.S41. Age is more strongly correlated with LBA $_{residual}$ when modelled with the adjustment (one-step) rather than the residual method (two-step). (A) MRi-Share, (B) HCP, (C) UKB, (D) Generation Scotland. Abbreviations: HCP = Human Connectome project, UKB = UK Biobank. Variance inflation is moderate to large for the one-step method. VIF was calculated with the car::vif() function in R for the adjustment method, but VIF cannot be estimated for the residual method because it only has one predictor variable.

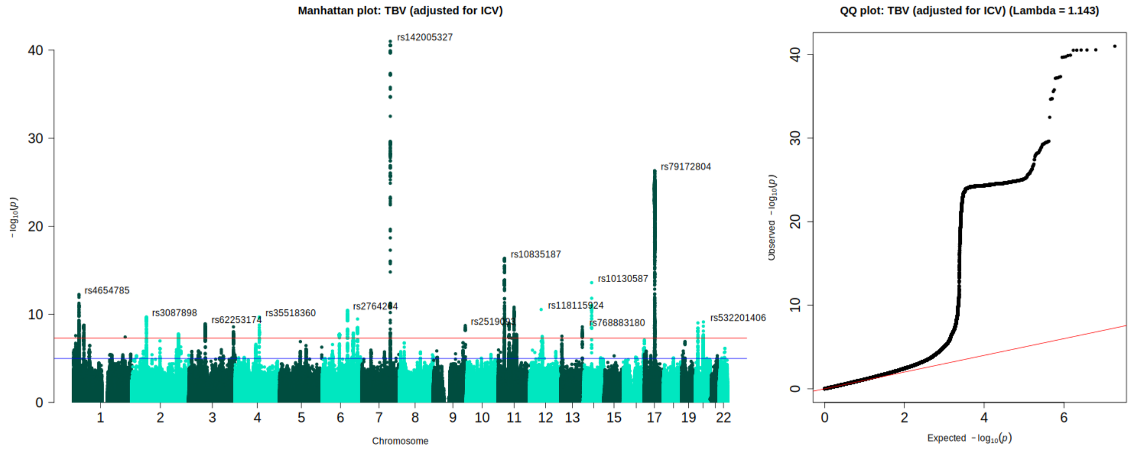

Fig.S42. Manhattan (left) and quantile-quantile plot (right) for lifetime brain atrophy ($N = 43{,}110$) inferred with the one-step residual method. Single nucleotide polymorphisms labelled in the Manhattan plot were the top GWAS hits. Lambda indicates the genomic inflation factor.

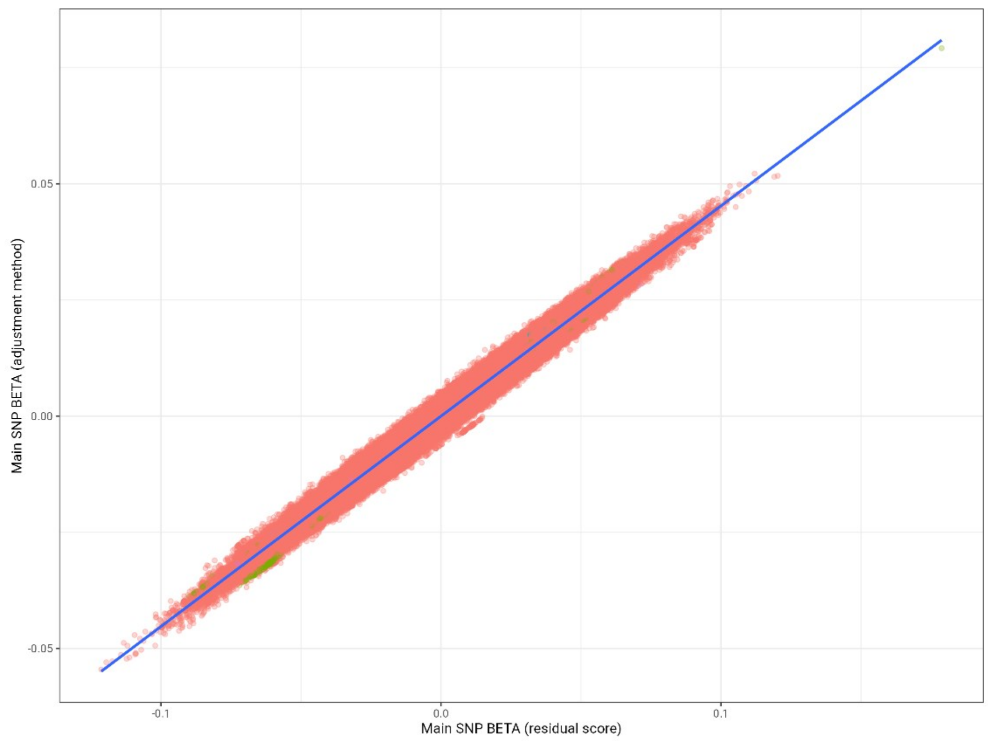

Fig.S43. Contrasting SNP beta effect sizes from LBA $_{residual}$ GWAS (*N* = 43,110) with SNP beta effect sizes from adjustment method GWAS (SNP filters: MAF > 0.01, INFO > 0.9, diallelic). Residual score was calculated with the two-step method, and adjustment method is the one-step procedure.

MRI processing robustness checks in LBC1936

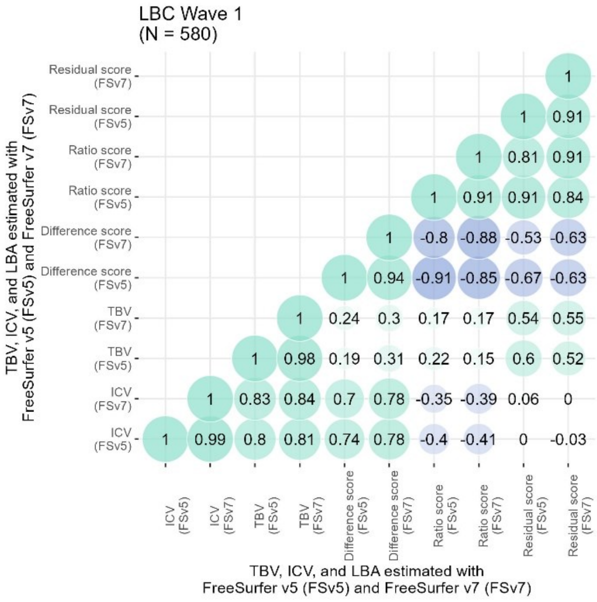

Fig. S44. Pearson's correlations across TBV, ICV and LBA measures that were obtained with FreeSurfer v5 (FSv5) and FreeSurfer v7 (FSv7). These correlations demonstrate that the variance

captured in TBV, ICV and lifetime brain atrophy (difference, ratio and residual score) are very similar when different versions of the FreeSurfer software were used for processing.

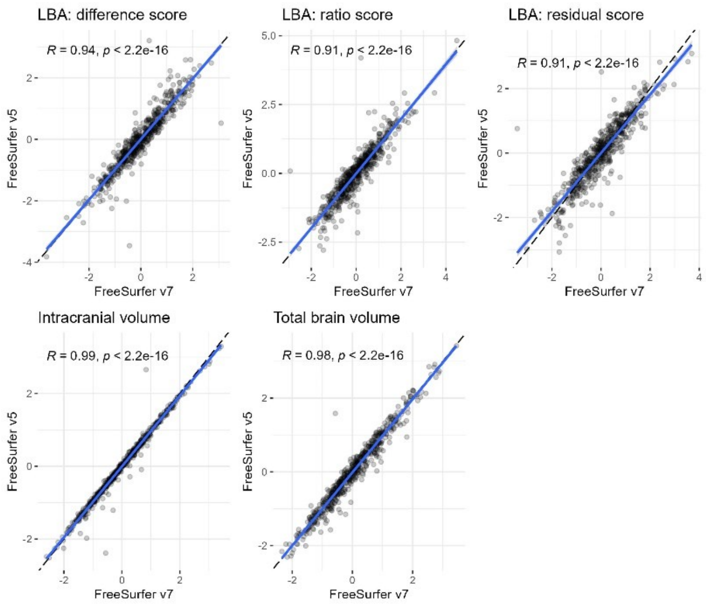

Fig. S45. Scatterplots to show linear associations between equivalent measures extracted from FreeSurfer v5 and FreeSurfer v7 ($N = 580$). These correlations demonstrate that the variance captured in TBV, ICV and lifetime brain atrophy (difference, ratio and residual score) are very similar when different versions of the FreeSurfer software were used for processing.

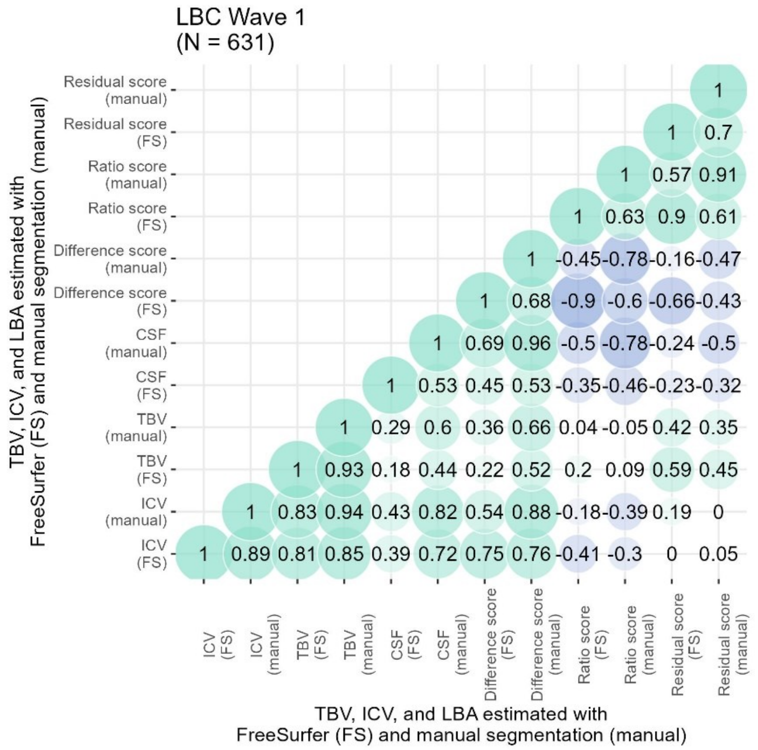

Fig. S46. Pearson's correlations across FreeSurferv5-based (FS) TBV, ICV and lifetime brain atrophy (difference, ratio and residual score) and manually segmented TBV, ICV and lifetime brain atrophy (difference, ratio and residual score). These correlations demonstrate that the variance

captured in TBV, ICV and lifetime brain atrophy are very similar when processing was done with the FreeSurfer software or by manual segmentation.

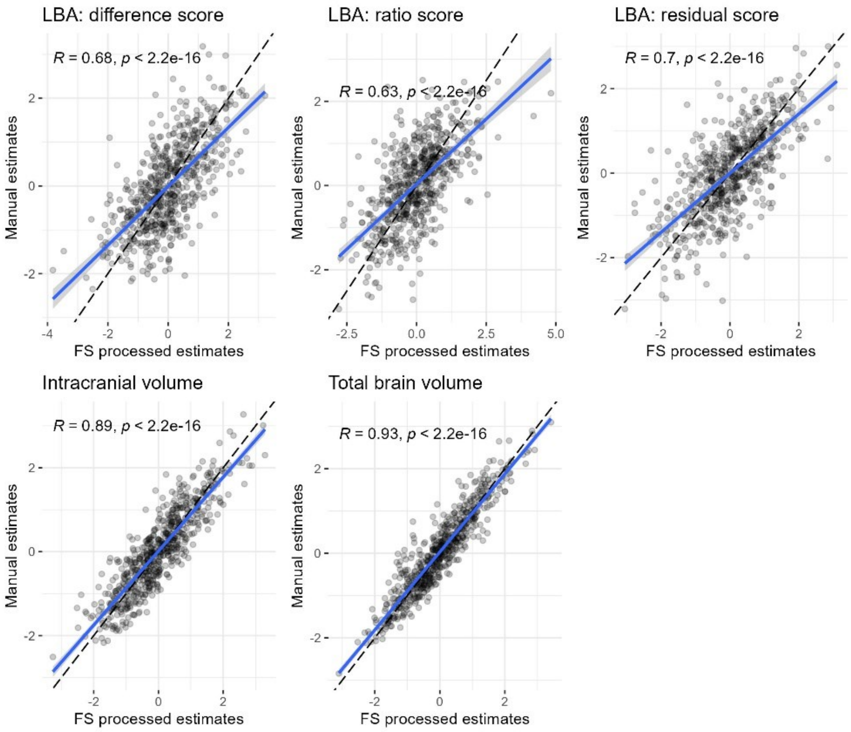

Fig. S47. Associations between equivalent measures extracted from FreeSurferv5 (FS)-based and manually segmented TBV and ICV estimates ($N = 631$). These correlations demonstrate that the variance captured in TBV, ICV and lifetime brain atrophy (difference, ratio and residual score) are very similar when processing was done with the FreeSurfer software or by manual segmentation.
