# Supplementary material for: Measurement characteristics and genome-wide correlates of lifetime brain atrophy estimated from a single MRI
Source: Nat Commun. 2025 Jul 21;16:6725. doi: 10.1038/s41467-025-61978-6 (PMC12280159; doi:10.1038/s41467-025-61978-6)
Supplement: Supplementary file 2 — Description of Addtional Supplementary Files [file 41467_2025_61978_MOESM2_ESM.pdf]

Description of Additional Supplementary Files

Supplementary Data 1. MRI scanner and acquisition information for each of the considered cohorts

References: (a) Van Essen et al. 2012 (1); (b) Tsuchida et al. 2021 (2); (c) https://www.fmrib.ox.ac.uk/ukbiobank/protocol/V4_23092014.pdf, Miller et al. 2016 (3); (d) https://lothian-birth-cohorts.ed.ac.uk/data-access-collaboration, Wardlaw et al. 2011 (4); (e) Habota et al. 2021 (5); (f) FreeSurfer reference: Fischl et al. 2002 (6), (g) https://www.fil.ion.ucl.ac.uk/spm/

Supplementary Data 2. Health outcome definitions considered in this study (UKB and LBC1936)

References: (a) Farrell et al., 2009 (7); (b) lavaan package Rosseel et al., 2012 (8); (c) Mullin et al., 2023 (9); (d) Kuo et al., 2020 (10); (e) Fried et al., 2001 (11); (f) Jiang et al., 2023 (12); (g) Fürtjes et al., 2022 (13); (h) Cox et al., 2019 (14); (i) Cole et al., 2018 (15); (j) Muñoz Maniega et al., 2019 (16)

Supplementary Data 3. Phenotypic analyses: Description and characterisation of the LBA phenotype

Supplementary Data 4. Cleaning of 45,515 available UKB participants with genotype data

Supplementary Data 5. Cleaning of 805,161 SNPs available in UKB genotype data (incl. sex chromosome)

Supplementary Data 6. Descriptive statistics of TBV, ICV and lifetime brain atrophy in younger (MRi-Share, HCP) and older adults (UKB, LBC1936)

Note: Same correlation between ICV and TBV (r = 0.93) when considering unrelated HCP individuals only (n = 326)

Supplementary Data 7. Sex-split correlations between LBA and longitudinally-observed atrophic changes

Supplementary Data 8. SNP-by-age interaction results

Supplementary Data 9. GWAS analysis adjusting for 8 genetic PCs only (instead of 40)

Supplementary Data 10. Genomic risk loci associated with the residual score defined by independent lead SNPs and maximum distance between their LD block (table copied from GenomicRiskLoci.txt output from FUMA)

Supplementary Data 11. Mapped genes from the residual score GWAS (table copied from genes.txt output from FUMA)

Supplementary Data 12. Genomic risk loci associated with the ratio score defined by independent lead SNPs and maximum distance between their LD block (table copied from GenomicRiskLoci.txt output from FUMA)

Supplementary Data 13. Mapped genes from the ratio score GWAS (table copied from genes.txt output from FUMA)

Supplementary Data 14. Genomic risk loci associated with the difference score defined by independent lead SNPs and maximum distance between their LD block (table copied from GenomicRiskLoci.txt output from FUMA)

Supplementary Data 15. Mapped genes from the difference score GWAS (table copied from genes.txt output from FUMA)

References

1. Van Essen DC, Ugurbil K, Auerbach E, Barch D, Behrens TEJ, Bucholz R, Chang A, Chen L, Corbetta M, Curtiss SW, Della Penna S, Feinberg D, Glasser MF, Harel N, Heath AC, Larson-Prior L, Marcus D, Michalareas G, Moeller S, Oostenveld R, Petersen SE, Prior F, Schlaggar BL, Smith SM, Snyder AZ, Xu J, Yacoub E. The Human Connectome Project: A data acquisition perspective. NeuroImage. 2012;62(4):2222-31.

2. Tsuchida A, Laurent A, Crivello F, Petit L, Joliot M, Pepe A, Beguedou N, Gueye M-F, Verrecchia V, Nozais V, Zago L, Mellet E, Debette S, Tzourio C, Mazoyer B. The MRi-Share database: brain imaging in a cross-sectional cohort of 1870 university students. Brain Structure and Function. 2021;226(7):2057-85.

3. Miller KL, Alfaro-Almagro F, Bangerter NK, Thomas DL, Yacoub E, Xu J, Bartsch AJ, Jbabdi S, Sotiropoulos SN, Andersson JLR, Griffanti L, Douaud G, Okell TW, Weale P, Dragonu I, Garratt S, Hudson S, Collins R, Jenkinson M, Matthews PM, Smith SM. Multimodal population brain imaging in the UK Biobank prospective epidemiological study. Nature Neuroscience. 2016;19(11):1523-36.

4. Wardlaw JM, Bastin ME, Valdés Hernández MC, Maniega SM, Royle NA, Morris Z, Clayden JD, Sandeman EM, Eadie E, Murray C, Starr JM, Deary IJ. Brain Aging, Cognition in Youth and Old Age and Vascular Disease in the Lothian Birth Cohort 1936: Rationale, Design and Methodology of the Imaging Protocol. International Journal of Stroke. 2011;6(6):547-59.

5. Habota T, Sandu A, Waiter G, McNeil C, Steele J, Macfarlane J, Whalley H, Valentine R, Younie D, Crouch N, Hawkins E, Hirose Y, Romaniuk L, Milburn K, Buchan G, Coupar T, Stirling M, Jagpal B, MacLennan B, Priba L, Harris M, Hafferty J, Adams M, Campbell A, MacIntyre D, Pattie A, Murphy L, Reynolds R, Elliot R, Penton-Voak I, Munafò M, Evans K, Seckl J, Wardlaw J, Lawrie S, Haley C, Porteous D, Deary I, Murray A, McIntosh A. Cohort profile for the STratifying Resilience and Depression Longitudinally (STRADL) study: A depression-focused investigation of Generation Scotland, using detailed clinical, cognitive, and neuroimaging assessments. Wellcome Open Research. 2021;4(185).

6. Fischl B, Salat DH, Busa E, Albert M, Dieterich M, Haselgrove C, Van Der Kouwe A, Killiany R, Kennedy D, Klaveness S. Whole brain segmentation: automated labeling of neuroanatomical structures in the human brain. Neuron. 2002;33(3):341-55.

7. Farrell C, Chappell F, Armitage PA, Keston P, MacLullich A, Shenkin S, Wardlaw JM. Development and initial testing of normal reference MR images for the brain at ages 65–70 and 75–80 years. European Radiology. 2009;19(1):177-83.

8. Rosseel Y. lavaan: An R package for structural equation modeling. Journal of statistical software. 2012;48:1-36.

9. Mullin DS, Stirland LE, Buchanan E, Convery C-A, Cox SR, Deary IJ, Giuntoli C, Greer H, Page D, Robertson E, Shenkin SD, Szalek A, Taylor A, Weatherdon G, Wilkinson T, Russ TC. Identifying dementia using medical data linkage in a longitudinal cohort study: Lothian Birth Cohort 1936. BMC psychiatry [Internet]. 2023 2023/05//; 23(1):[303 p.].

10. Kuo C-L, Pilling LC, Atkins JL, Kuchel GA, Melzer D. ApoE e2 and aging-related outcomes in 379,000 UK Biobank participants. Aging (Albany NY). 2020;12(12):12222.

11. Fried LP, Tangen CM, Walston J, Newman AB, Hirsch C, Gottdiener J, Seeman T, Tracy R, Kop WJ, Burke G, McBurnie MA. Frailty in Older Adults: Evidence for a Phenotype. The Journals of Gerontology: Series A. 2001;56(3):M146-M57.

12. Jiang R, Noble S, Sui J, Yoo K, Rosenblatt M, Horien C, Qi S, Liang Q, Sun H, Calhoun VD, Scheinost D. Associations of physical frailty with health outcomes and brain structure in 483 033 middle-aged and older adults: a population-based study from the UK Biobank. The Lancet Digital Health. 2023;5(6):e350-e9.
13. Fürtjes AE, Coleman JRI, Tyrrell J, Lewis CM, Hagenaars SP. Associations and limited shared genetic aetiology between bipolar disorder and cardiometabolic traits in the UK Biobank. Psychological Medicine. 2022;52(16):4039-48.
14. Cox SR, Lyall DM, Ritchie SJ, Bastin ME, Harris MA, Buchanan CR, Fawns-Ritchie C, Barbu MC, de Nooij L, Reus LM, Alloza C, Shen X, Neilson E, Alderson HL, Hunter S, Liewald DC, Whalley HC, McIntosh AM, Lawrie SM, Pell JP, Tucker-Drob EM, Wardlaw JM, Gale CR, Deary IJ. Associations between vascular risk factors and brain MRI indices in UK Biobank. European Heart Journal. 2019;40(28):2290-300.
15. Cole JH, Ritchie SJ, Bastin ME, Valdés Hernández MC, Muñoz Maniega S, Royle N, Corley J, Pattie A, Harris SE, Zhang Q, Wray NR, Redmond P, Marioni RE, Starr JM, Cox SR, Wardlaw JM, Sharp DJ, Deary IJ. Brain age predicts mortality. Molecular Psychiatry. 2018;23(5):1385-92.
16. Muñoz Maniega S, Meijboom R, Chappell FM, Valdés Hernández MdC, Starr JM, Bastin ME, Deary IJ, Wardlaw JM. Spatial Gradient of Microstructural Changes in Normal-Appearing White Matter in Tracts Affected by White Matter Hyperintensities in Older Age. Frontiers in Neurology. 2019;10.
