# Supplementary material for: Measurement characteristics and genome-wide correlates of lifetime brain atrophy estimated from a single MRI
Source: Nat Commun. 2025 Jul 21;16:6725. doi: 10.1038/s41467-025-61978-6 (PMC12280159; doi:10.1038/s41467-025-61978-6)
Supplement: Supplementary file 4 — Reporting Summary [file 41467_2025_61978_MOESM4_ESM.pdf]

nature portfolio

Corresponding author(s): Anna E. Fürtjes

Last updated by author(s): 19/03/2025

Reporting Summary

Nature Portfolio wishes to improve the reproducibility of the work that we publish. This form provides structure for consistency and transparency in reporting. For further information on Nature Portfolio policies, see our Editorial Policies and the Editorial Policy Checklist.

Statistics

For all statistical analyses, confirm that the following items are present in the figure legend, table legend, main text, or Methods section.

n/a	Confirmed	
☐	☒	The exact sample size (*n*) for each experimental group/condition, given as a discrete number and unit of measurement
☐	☒	A statement on whether measurements were taken from distinct samples or whether the same sample was measured repeatedly
☐	☒	The statistical test(s) used AND whether they are one- or two-sided<br>*Only common tests should be described solely by name; describe more complex techniques in the Methods section.*
☐	☒	A description of all covariates tested
☐	☒	A description of any assumptions or corrections, such as tests of normality and adjustment for multiple comparisons
☐	☒	A full description of the statistical parameters including central tendency (e.g. means) or other basic estimates (e.g. regression coefficient) AND variation (e.g. standard deviation) or associated estimates of uncertainty (e.g. confidence intervals)
☐	☒	For null hypothesis testing, the test statistic (e.g. *F*, *t*, *r*) with confidence intervals, effect sizes, degrees of freedom and *P* value noted<br>*Give P values as exact values whenever suitable.*
☒	☐	For Bayesian analysis, information on the choice of priors and Markov chain Monte Carlo settings
☒	☐	For hierarchical and complex designs, identification of the appropriate level for tests and full reporting of outcomes
☐	☒	Estimates of effect sizes (e.g. Cohen's *d*, Pearson's *r*), indicating how they were calculated

Our web collection on statistics for biologists contains articles on many of the points above.

Software and code

Policy information about availability of computer code

Data collection	Data analysed to produce results presented in this paper are all described in the Supplementary Methods, including references to central papers describing the resource and its collection.
Data analysis	All analyses relied on open source software, and code written for this project is summarised in https://annafurtjes.github.io/BrainAtrophy_Genetics/<br>Used software: R (v4.2.2), GCTA (v1.94.1), REGENIE (v3.4)

For manuscripts utilizing custom algorithms or software that are central to the research but not yet described in published literature, software must be made available to editors and reviewers. We strongly encourage code deposition in a community repository (e.g. GitHub). See the Nature Portfolio guidelines for submitting code & software for further information.

Data

Policy information about availability of data

All manuscripts must include a data availability statement. This statement should provide the following information, where applicable:

- Accession codes, unique identifiers, or web links for publicly available datasets
- A description of any restrictions on data availability
- For clinical datasets or third party data, please ensure that the statement adheres to our policy

All cohorts are openly available. HCP https://www.humanconnectome.org/ ; MRi-Share https://doi.org/10.5061/dryad.q573n5tj2 ; UKB https://

www.ukbiobank.ac.uk/enable-your-research/apply-for-access ; LBC1936 https://lothian-birth-cohorts.ed.ac.uk/data-access-collaboration ; STRADL is available upon request https://wellcomeopenresearch.org/articles/4-185. GWAS summary data are available under https://doi.org/10.5281/zenodo.14860747.

Research involving human participants, their data, or biological material

Policy information about studies with human participants or human data. See also policy information about sex, gender (identity/presentation), and sexual orientation and race, ethnicity and racism.

Reporting on sex and gender	Primary analyses were repeated in males and females separately to confirm that results were not dependent upon biological sex.
Reporting on race, ethnicity, or other socially relevant groupings	Phenotypic and genetic analyses were conducted in European-only ancestry. We did not have sufficient power in the current sample to analyse specific non-European ancestry. This work is a pre-cursor paper to confirm the validity of the LBA phenotype, which will now be followed up by multi-ancestry meta-analyses (efforts already underway).
Population characteristics	ICV, TBV and LBA phenotypes across all cohorts are described in Table S6. GWAS nuisance covariates in this study were sex, acquisition site, acquisition time, scan positions (x,y,z), genetic principal components, genotyping array, genotyping batch.
Recruitment	All cohort were recruited on a voluntary basis, and their respective recruitment approaches are outlined in the Supplementary Methods.
Ethics oversight	Each of the cohorts had their own ethical approval described in their respective papers linked in the Supplementary Methods. We obtained approvals where required to access and analyse the data for this project in accordance with data access regulations.All cohorts followed appropriate ethical regultions and obtained informed consent from their participants.

Note that full information on the approval of the study protocol must also be provided in the manuscript.

Field-specific reporting

Please select the one below that is the best fit for your research. If you are not sure, read the appropriate sections before making your selection.

☐ Life sciences ☒ Behavioural & social sciences ☐ Ecological, evolutionary & environmental sciences

For a reference copy of the document with all sections, see nature.com/documents/nr-reporting-summary-flat.pdf

Behavioural & social sciences study design

All studies must disclose on these points even when the disclosure is negative.

Study description	Lifetime brain atrophy is a continuous phenotype which was standardised (mean = 0, sd = 1) and was analysed with different linear models: Pearson's correlations, simple and multiple linear regression, mixed linear model (SNP-level analyses)
Research sample	HCP - young adult twins in the US, MRi-Share - University Students in Bordeaux, France, UKB - population of United Kingdom, LBC1936 - Participants in the Lothian area born in 1936 and participated in Scottish Mental Survey in 1947, STRADL - Population of Scotland
Sampling strategy	This study analysed observational studies (cross-sectional and longitudinal), and we used samples of convenience in this context.
Data collection	See Supplementary Methods for MRI scanner & acquisition information which differs for each cohort.
Timing	See Supplementary Methods for data collection protocols described in linked references.
Data exclusions	Participants were excluded if TBV > ICV, or LBA > |10| SDs, or if genetic data did not meet quality criteria, or if covariate info was unavailable
Non-participation	See Supplementary Methods for data collection protocols described in linked references.
Randomization	Data was not randomised as this study does not include clinical trials.

Reporting for specific materials, systems and methods

We require information from authors about some types of materials, experimental systems and methods used in many studies. Here, indicate whether each material, system or method listed is relevant to your study. If you are not sure if a list item applies to your research, read the appropriate section before selecting a response.

Materials & experimental systems

n/a	Involved in the study
☒	☐ Antibodies
☒	☐ Eukaryotic cell lines
☒	☐ Palaeontology and archaeology
☒	☐ Animals and other organisms
☒	☐ Clinical data
☒	☐ Dual use research of concern
☒	☐ Plants

Methods

n/a	Involved in the study
☒	☐ ChIP-seq
☒	☐ Flow cytometry
☐	☒ MRI-based neuroimaging

Plants

Seed stocks	NA
Novel plant genotypes	NA
Authentication	NA

Magnetic resonance imaging

Experimental design

Design type	Structural neuroimaging (T1)
Design specifications	Cross-sectional (all cohorts) & repeated assessment with two measured time points (LBC1936 & UKB)
Behavioral performance measures	See Supplementary Methods (Section 2.1.4) for list of all behavioral variables and their corresponding variable names in UKB and LBC1936

Acquisition

Imaging type(s)	Structural neuroimaging (T1)
Field strength	1.5T (LBC1936, UKB); 3T (MRi-Share, HCP, STRADL)
Sequence & imaging parameters	See Supplementary Table S1 for imaging parameters listed for each cohort
Area of acquisition	HCP: Washington, MRi-Share: Bordeaux, France, UKB: Manchester, Newcastle, and Reading, UK, STRADL: Aberdeen, Dundee, UK, LBC1936: Edinburgh, UK

Diabetes MRI ☐ Used ☒ Not used

Preprocessing

Preprocessing software	FreeSurfer, cross-sectional processing stream (different versions, see Table S1), longitudinal processing stream also used in LBC1936 and UKB. Supplementary Method section 2.1.3 compares FreeSurfer v5 with FreeSurfer v7 and manual segmentation in the LBC1936.
Normalization	Performed by cohort analysts, FreeSurfer default settings, cross-sectional and longitudinal processing stream (different versions, see Table S1)
Normalization template	fsaverage template (cross-sectional), within-individual average template across all time points for a participant (longitudinal processing)
Noise and artifact removal	FreeSurfer recon-all includes some noise and artifact removal steps (skull stripping, motion correction, intensity normalisation)
Volume censoring	NA

Statistical modeling & inference

Model type and settings	LBA inferred with difference (ICV - TBV), ratio (TBV / ICV), and regression-residual method (TBV ~ ICV)
Effect(s) tested	Different linear models: Pearson's correlations, multiple regression, mixed linear model (SNP-level analyses)

Specify type of analysis: ☒ Whole brain ☐ ROI-based ☐ Both

Statistic type for inference (See Eklund et al. 2016)	NA (No voxel- or cluster-wise analysis)
Correction	Bonferroni correction for multiple testing

Models & analysis

n/a	Involved in the study
☒	☐ Functional and/or effective connectivity
☒	☐ Graph analysis
☐	☒ Multivariate modeling or predictive analysis

Multivariate modeling and predictive analysis	Different linear models: Pearson's correlations, multiple regression, mixed linear model (SNP-level analyses)
